# The exercise-microbiota-queuine-tRNA axis in Parkinson's disease: evidence, uncertainties, and experimental priorities

**DOI:** 10.3389/fnins.2026.1854892

**Published:** 2026-06-11

**Authors:** Yang Zhou, Tao Jiang

**Affiliations:** 1 Guangxi Science and Technology Normal University, Laibin, China; 2 Guangxi University, Nanning, China

**Keywords:** evidence hierarchy, exercise, gut microbiota, mitochondrial translational stress, Parkinson's disease, precision medicine, Q-tRNA modification, queuine

## Abstract

Parkinson's disease (PD) is a multisystem neurodegenerative disorder characterized by progressive nigrostriatal dopaminergic degeneration, α-synuclein aggregation, mitochondrial dysfunction, oxidative stress, and neuroinflammatory remodeling. Although these mechanisms have been extensively investigated, how systemic metabolic and microbiota-derived signals intersect with neuronal translational control remains incompletely understood. Queuosine (Q) modification of tRNAs is a distinctive RNA modification because its precursor, queuine, is not synthesized *de novo* by mammalian cells but is acquired from diet and gut microbial metabolism. Emerging evidence indicates that Q-tRNA modification can influence codon decoding, translational speed, proteostasis, oxidative stress responses, and mitochondrial function, but direct evidence linking Q-tRNA dysregulation to PD remains limited. In this narrative review, we propose a conceptual and hypothesis-generating framework in which the microbiota-queuine-Q-tRNA modification axis may contribute to neuronal translational buffering and stress adaptation in PD. We distinguish established mechanisms, emerging evidence, and speculative links, emphasizing that the complete causal chain from exercise-induced microbiota remodeling to altered queuine availability, Q-tRNA modification, mitochondrial translational recalibration, and dopaminergic neuroprotection has not yet been experimentally demonstrated. We further discuss tRNA-derived fragments (tRFs) as candidate biomarkers and potential effector molecules in PD-associated translational stress, neuroinflammation, and intercellular RNA communication. Finally, we outline experimental priorities for validating this model, including direct Q-tRNA profiling in PD tissues and biofluids, exercise-intervention studies in PD models, microbiota/queuine manipulation, and mechanistic testing of circulating RNA carrier transport across the blood-brain barrier. This framework does not establish a new pathogenic pathway, but provides a structured roadmap for investigating how exercise, microbial metabolism, and RNA modification biology may converge on selective neuronal vulnerability in PD.

## 1 Introduction

Parkinson's disease (PD) is the second most common neurodegenerative disorder after Alzheimer's disease (Poewe et al., 2017; Paldor et al., 2023). Its main pathological hallmarks include the progressive loss of dopaminergic neurons in the substantia nigra pars compacta (SNpc) and the abnormal aggregation of α-synuclein (α-syn) in Lewy bodies (Poewe et al., 2017; Paldor et al., 2023; Spillantini et al., 1997; Surmeier et al., 2017). Although the clinical and molecular features of PD have been extensively studied, its exact pathogenesis remains partially undetermined. Previous evidence has mainly centered on protein misfolding, mitochondrial impairment, oxidative stress, and neuroinflammation (Surmeier et al., 2017; Lashuel et al., 2013; Schapira, 2007; Wong and Krainc, 2017; Bose and Beal, 2016). Recently, a growing body of evidence has revealed that these pathological processes are not independent of each other, but rather form a complex interactive network at the levels of cellular metabolism, protein homeostasis, and transcriptional regulation, collectively driving the progression of neurodegeneration (Surmeier et al., 2017; Lashuel et al., 2013; Schapira, 2007; Wong and Krainc, 2017; Bose and Beal, 2016; Sampson et al., 2016; Romano et al., 2021).

Among the molecular processes that may bridge mitochondrial dysfunction, proteostatic stress, and neuronal vulnerability, translational homeostasis has received increasing attention. Protein synthesis is shaped not only by mRNA abundance, but also by codon-decoding kinetics, ribosomal pausing, aminoacyl-tRNA charging, tRNA modifications, and stress-responsive translational reprogramming (Kirchner and Ignatova, 2015; Nedialkova and Leidel, 2015; Mick et al., 2020; Pakos-Zebrucka et al., 2016). These layers of regulation are particularly relevant to neurons with high energetic and proteostatic demands, such as substantia nigra pars compacta (SNpc) dopaminergic neurons (Surmeier et al., 2017; Ni and Ernst, 2022; Watanabe et al., 2024). However, whether altered tRNA biology directly contributes to PD pathogenesis remains an emerging question rather than an established mechanism.

Queuosine (Q) modification represents a biologically distinctive tRNA modification because its precursor, queuine, depends on diet and microbial metabolism rather than mammalian *de novo* synthesis (Ehrenhofer-Murray, 2025; Tuorto et al., 2018; Rashad, 2025; Dong et al., 2025). Q modification occurs at the wobble position of selected tRNAs and can influence codon decoding, translational speed, and protein-folding homeostasis (Tuorto et al., 2018; Dong et al., 2025). Experimental work has shown that nutritionally determined Q-tRNA levels regulate the translation speed of Q-decoded codons and interact with other tRNA modifications, such as Dnmt2-dependent methylation of tRNA^∧^Asp (Tuorto et al., 2018). In parallel, emerging studies suggest that queuine may influence mitochondrial function in specific disease contexts, although direct evidence in PD remains insufficient (Lin et al., 2024).

PD is also increasingly recognized as a disorder in which gut-brain communication, systemic inflammation, and peripheral metabolic remodeling may contribute to disease heterogeneity (Sampson et al., 2016; Romano et al., 2021; Peters et al., 2025; Scheperjans et al., 2015; Bedarf et al., 2017). Exercise is one of the most promising non-pharmacological interventions for PD (Ahlskog, 2018; Petzinger et al., 2013; Pizzo et al., 2013), but its molecular effects are heterogeneous and not fully explained by improvements in muscle strength or motor practice alone. We therefore propose, rather than establish, an exercise-microbiota-queuine-tRNA modification framework. In this model, exercise may influence gut microbial metabolism, circulating RNA carriers, and systemic metabolic signals (Wei et al., 2025; Oliveira et al., 2018; Karvinen et al., 2023; Denham and Spencer, 2020; Allen et al., 2018; Mailing et al., 2019); these changes could, in principle, modulate queuine availability, Q-tRNA modification, tRNA-derived fragments, and neuronal stress adaptation. Because several links in this chain remain indirect or untested, this review explicitly separates established mechanisms, emerging evidence, and hypothesis-generating links.

On the basis of these converging but uneven lines of evidence, this review proposes the exercise-microbiota-queuine-tRNA modification axis as a conceptual framework rather than a validated causal pathway. The framework rests on three evidence layers: established biology, including microbial/dietary queuine supply and QTRT1/QTRT2-mediated Q-tRNA formation; emerging evidence, including tRF alterations in PD biofluids and exercise-induced remodeling of gut microbiota and circulating RNA carriers; and speculative but testable links, including exercise-induced modulation of Q-tRNA modification and consequent protection of SNpc dopaminergic neurons. This evidence-stratified approach is used throughout the review to avoid presenting hypothesis-generating mechanisms as established facts.

## 2 Materials and methods

This article was designed as a narrative review with a hypothesis-generating and evidence-mapping orientation. It was not intended to be a systematic review or meta-analysis. Therefore, no formal protocol was registered, no quantitative evidence synthesis was performed, and no risk-of-bias tool was applied to individual studies. To improve transparency and reduce selection bias, the literature search, inclusion logic, and evidence-stratification strategy are described below. The reporting approach was informed by recommendations for improving the transparency of narrative reviews and by the Scale for the Assessment of Narrative Review Articles (SANRA) (Baethge et al., 2019).

### 2.1 Literature search strategy

Literature was searched in PubMed, Web of Science, Scopus, and Google Scholar. The primary search period covered studies published from January 2000 to December 2025, with particular attention to evidence published from 2020 to 2025 because tRNA-derived fragments, Q-tRNA biology, and exercise-responsive extracellular RNA carriers have rapidly expanded during this period. Additional references were identified by backward citation searching from relevant reviews and key mechanistic studies.

Representative PubMed search strings included the following:

(1) (“Parkinson disease” OR “Parkinson's disease” OR PD) AND (“tRNA modification” OR “queuosine” OR “queuine” OR “Q-tRNA” OR QTRT1 OR QTRT2)
(2) (“Parkinson disease” OR “Parkinson's disease”) AND (“tRNA-derived fragments” OR tRF OR tsRNA OR tiRNA OR angiogenin)
(3) (“queuine” OR “queuosine” OR “Q-tRNA”) AND (“translation” OR “codon decoding” OR “proteostasis” OR “mitochondria” OR “oxidative stress”)
(4) (“exercise” OR “aerobic exercise” OR “treadmill training”) AND (“Parkinson disease” OR “Parkinson's disease”) AND (“gut microbiota” OR microbiome OR metabolomics OR “extracellular vesicles” OR “small RNA”)
(5) (“extracellular vesicles” OR HDL OR “high-density lipoprotein”) AND (“small RNA” OR “tRNA-derived RNA” OR tRF OR microRNA) AND (“blood-brain barrier” OR brain OR neurodegeneration)

### 2.2 Inclusion and exclusion criteria

Studies were included if they met at least one of the following criteria: (1) original experimental or clinical studies investigating Q-tRNA modification, queuine metabolism, QTRT1/QTRT2, or related tRNA modification pathways; (2) studies examining tRFs/tsRNAs/tiRNAs in PD, neurodegeneration, cellular stress, extracellular vesicles, or biofluids; (3) studies assessing mitochondrial translational stress, integrated stress response, tRNA wobble modifications, or proteostatic stress relevant to neuronal vulnerability; (4) human or animal studies evaluating exercise-induced changes in PD-related motor/non-motor outcomes, gut microbiota, circulating metabolites, extracellular vesicles, or small RNA cargo; (5) reviews or methodological articles used to contextualize narrative review reporting, extracellular RNA biology, gut-brain communication, or biomarker translation.

Studies were excluded if they were unrelated to PD, tRNA biology, queuine/Q-tRNA modification, exercise, gut microbiota, mitochondrial stress, or extracellular RNA signaling; if they were duplicate reports; if they lacked sufficient methodological detail; or if they made mechanistic claims without experimental or clinical support. Non-peer-reviewed preprints were considered only when directly relevant to an emerging mechanism and were explicitly labeled as non-peer-reviewed and hypothesis-generating.

### 2.3 Evidence stratification

Because the proposed exercise-microbiota-queuine-tRNA modification axis spans multiple fields and contains several untested links, evidence was stratified into three levels throughout the manuscript: Established mechanisms: mechanisms supported by multiple peer-reviewed studies or well-characterized biochemical pathways, such as bacterial/dietary queuine supply, QTRT1/QTRT2-mediated incorporation of queuine into selected tRNAs, and ANG-mediated tRNA cleavage under cellular stress. Emerging evidence: findings supported by limited but peer-reviewed experimental, clinical, or biomarker studies, such as PD-associated tRF signatures in blood/CSF/brain tissue, exercise-associated gut microbiota remodeling in PD, and exercise-responsive changes in extracellular RNA carriers. Hypothesis-generating links: mechanistic connections that are biologically plausible but not yet directly demonstrated, such as exercise-induced changes in Q-tRNA modification in PD, microbiota-dependent modulation of queuine/preQ1 competition in PD, and Q-tRNA-mediated protection of SNpc dopaminergic neurons after exercise.

### 2.4 Limitations of the review methodology

As a narrative review, this article does not provide exhaustive literature coverage, pooled effect estimates, or formal risk-of-bias assessment. The conclusions may therefore be influenced by publication bias, study-selection bias, and the uneven maturity of evidence across RNA biology, microbiome science, and exercise neuroscience. To mitigate these limitations, we avoid definitive causal language when evidence is indirect, identify non-peer-reviewed or single-study evidence explicitly, and provide a dedicated evidence map and experimental-priority section to clarify which links are established, emerging, or speculative.

### 2.5 Evidence hierarchy for the proposed exercise-microbiota-queuine-tRNA modification axis

The proposed axis should be interpreted as a layered framework rather than a continuous experimentally verified pathway. Table 1 summarizes the current strength of evidence for each step and identifies the major unresolved questions. This evidence map is intended to prevent overinterpretation and to guide experimental validation.

**Table 1:** Evidence hierarchy and unresolved links in the proposed exercise-microbiota-queuine-tRNA modification axis.

Proposed link	Evidence level	Current support	Main limitation	Interpretation
Gut microbiota and diet provide queuine for host Q-tRNA modification (Ehrenhofer-Murray, 2025; Tuorto et al., 2018; Rashad, 2025; Dong et al., 2025)	Established mechanism	Mammals cannot synthesize queuine *de novo*; queuine is obtained from diet and microbial metabolism and incorporated into selected tRNAs by QTRT1/QTRT2	Species-specific differences in microbial queuine production and host salvage remain incompletely mapped	Strong biochemical foundation
Q-tRNA modification influences codon decoding and proteostasis (Tuorto et al., 2018; Dong et al., 2025)	Established to emerging mechanism	Q-tRNA levels regulate translation speed of Q-decoded codons and interact with Dnmt2-dependent tRNA methylation (Tuorto et al., 2018)	Most evidence comes from non-neuronal or non-PD models	Relevant to translational quality control, but PD-specific evidence is limited
Q-tRNA modification may intersect with mitochondrial stress adaptation (Lin et al., 2024; Wei et al., 2025; Müller et al., 2019)	Emerging and partly hypothesis-generating	Queuine supplementation can improve mitochondrial dysfunction caused by mt-tRNA^∧^Asn variants; non-peer-reviewed preprint evidence suggests dynamic Q-tRNA remodeling under mitochondrial stress (Lin et al., 2024)	The strongest mitochondrial stress/Q-tRNA dataset remains limited and not fully validated in PD	Should be framed as a candidate regulatory node, not a master regulator
PD is associated with altered tRF/tsRNA profiles (Paldor et al., 2023; Madrer et al., 2025; Baindoor et al., 2024; Magee et al., 2019)	Emerging evidence	PD-related tRF signatures have been reported in CSF, blood, and substantia nigra; RGTTCRA-tRFs and MT-tRF shifts may have biomarker potential (Paldor et al., 2023; Madrer et al., 2025)	Independent replication, longitudinal validation, and mechanistic interpretation remain incomplete	Useful for biomarker-oriented stratification
tRFs can act as effector molecules in PD-related neuroinflammation (Dong et al., 2025)	Emerging evidence	EV-associated tRF-02514 was reported to promote microglial pyroptosis and neuronal loss by targeting ATG5 in PD models (Dong et al., 2025)	Evidence is still early and focused on selected tRF species	Candidate effector mechanism
Exercise remodels gut microbiota and circulating RNA carriers (Wei et al., 2025; Oliveira et al., 2018; Karvinen et al., 2023; Denham and Spencer, 2020; Allen et al., 2018; Mailing et al., 2019)	Emerging evidence	Exercise can alter gut microbiota in PD and change EV/HDL-associated small RNA cargo, including tRNA-derived small RNAs (Wei et al., 2025; Karvinen et al., 2023)	Exercise protocols, disease stage, medication status, and microbiota baseline vary substantially	Plausible upstream modulator
Exercise alters Q-tRNA modification in PD	Hypothesis-generating	No direct *in vivo* evidence currently demonstrates that exercise changes Q-tRNA levels in PD brain or biofluids	Requires targeted LC-MS/MS, APB northern blot, or tRNA modification sequencing after exercise intervention	Major experimental priority
Exercise-microbiota-queuine-Q-tRNA modulation may influence SNpc dopaminergic neuronal vulnerability	Hypothesis-generating	Indirect support comes from exercise neuroprotection, microbiota remodeling, and Q-tRNA biology	The complete causal chain has not been demonstrated	Hypothesis-generating link requiring stepwise validation

## 3 Findings

### 3.1 The physiological basis of the queuine-Q-tRNA modification axis

#### 3.1.1 The source of queuine: a bacteria-dependent, host-utilizable nucleobase

Queuine/queuosine (Q) is one of the few tRNA-modification-related metabolites that exemplify microbiota-host co-metabolism. Q is positioned at the wobble position (position 34) of notable tRNA anticodons. Its complete biosynthetic pathway exists only in bacteria, while eukaryotes lack the ability for *de novo* synthesis, making them “nutritionally/microbiota-dependent” on queuine (Ehrenhofer-Murray, 2025; Rashad, 2025). This feature distinguishes Q modification from many host-derived RNA modifications and provides a plausible molecular link between diet, gut microbial metabolism, and host translational regulation. Conceptually, queuine may act not only as a modification substrate but also as a potential molecular interface through which microbial and nutritional states influence host translation. In mammals, queuine is mainly obtained from dietary sources and gut microbial metabolism and is subsequently salvaged by host cells for Q-tRNA modification (Ehrenhofer-Murray, 2025; Tuorto et al., 2018; Rashad, 2025; Dong et al., 2025). Thus, Q modification levels may reflect not only host enzymatic capacity but also external nutritional input and gut microbial metabolism. It has been indicated in reviews that this “gut-tRNA axis” can connect gut health and the metabolic environment with the tissue translatome/proteome state (Rashad, 2025). In this regard, the queuine-Q-tRNA axis provides a plausible entry point for investigating how gut-brain communication, exercise-microbiota interactions, and neurodegenerative stress may intersect with translational homeostasis.

#### 3.1.2 The Q-tRNA modification process driven by QTRT1/QTRT2

In eukaryotic cells, Q modification is mediated by the eukaryotic tRNA-guanine transglycosylase (eTGT) complex, which consists of the enzymatically active QTRT1 and the non-catalytic QTRT2, critical for substrate recognition and RNA binding (Rashad, 2025; Fergus et al., 2021; Johannsson et al., 2018; Sievers et al., 2024). This reaction involves recognition of specific GUN-anticodon tRNAs by the QTRT1/QTRT2 complex and replacement of guanine at position 34 with queuine through a base-exchange reaction, thereby generating Q-modified tRNAs (Fergus et al., 2021; Johannsson et al., 2018; Sievers et al., 2024). Unlike bacterial TGT enzymes, the eukaryotic TGT complex forms a heterodimer, suggesting more specialized substrate recognition and potentially greater sensitivity to cellular context. In mammals, Q modification primarily occurs in four GUN-anticodon tRNAs: tRNA^∧^Asp, tRNA^∧^Asn, tRNA^∧^His, and tRNA^∧^Tyr (Ehrenhofer-Murray, 2025; Fergus et al., 2021; Johannsson et al., 2018; Sievers et al., 2024). Evidence from structural biology indicates that QTRT2, despite being non-catalytic, participates in substrate tRNA binding and proper positioning of the anticodon stem-loop (Fergus et al., 2021; Sievers et al., 2024).

Therefore, QTRT1/QTRT2 should be viewed not simply as a catalytic-plus-accessory pair, but as a functional complex that coordinates substrate recognition, anticodon-loop positioning, and catalysis. This complexity at the molecular level serves as a basis for future studies on how Q modification affects codon interpretation and selective translation during stress.

#### 3.1.3 How Q-tRNA impacts codon recognition, translational accuracy, and proteostasis

Q modification is physiologically important because it occurs at the wobble position of the anticodon, where it can influence codon-anticodon pairing dynamics, decoding speed, and fidelity (Ehrenhofer-Murray, 2025; Tuorto et al., 2018; Rashad, 2025). Previous studies have shown that Q modification can influence the decoding balance of NAU/NAC synonymous codons and modulate the translation speed of Q-dependent codons; in the absence of Q, ribosomal pausing at corresponding codons increases and near-cognate decoding may also be affected (Tuorto et al., 2018). This indicates that altering Q affects not just the efficiency of one codon, but also influences codon optimality, the rate of translational elongation, and local translational accuracy more broadly. Q modification may also interact functionally with other tRNA modifications. For example, nutritionally determined Q-tRNA levels can promote Dnmt2-mediated methylation of tRNA^∧^Asp at C38, thereby influencing translational programs and proteostasis (Tuorto et al., 2018). Research by Tuorto et al. demonstrated that a lack of queuine alters the speed of translation at the codon level and leads to the buildup of misfolded proteins, triggering endoplasmic reticulum stress and the unfolded protein response (UPR; Tuorto et al., 2018). Thus, Q-tRNA may influence proteostasis not only by altering overall translational output but also by shaping codon-specific translational quality control.

Taken together, Q modification provides a biologically plausible, but not yet disease-proven, entry point for linking codon-decoding kinetics, protein-folding stress, and translational imbalance in neurodegenerative contexts. In PD specifically, direct profiling of Q-tRNA modification in vulnerable brain regions and patient biofluids is still required before Q deficiency can be considered a disease mechanism.

#### 3.1.4 The possible intersection between Q-tRNA modification and mitochondrial function

Although Q modification was initially characterized as an anticodon-loop modification of cytoplasmic tRNAs, accumulating evidence suggests that queuine/Q-tRNA biology may also intersect with mitochondrial function, oxidative-stress adaptation, and translational quality control. Mechanistically, Q modification can influence codon-decoding kinetics, ribosomal elongation speed, and protein-folding homeostasis, thereby providing a plausible route through which Q-tRNA status may affect stress-sensitive proteomes (Tuorto et al., 2018; Müller et al., 2019). Importantly, Q modification is not restricted to cytoplasmic tRNAs. A comprehensive chemical analysis of human mitochondrial tRNAs identified Q34 in mitochondrial tRNAs for Asp, His, Asn, and Tyr, and showed that QTRT1 and QTRT2 are required for Q34 formation in these mt-tRNAs (Suzuki et al., 2020). This finding provides a direct molecular basis for considering QTRT-dependent queuosine biology in the context of mitochondrial translation.

Functional evidence further supports, but does not yet prove in PD, a link between queuine biology and mitochondrial homeostasis. In a mitochondrial disease context, queuine supplementation was reported to improve mt-tRNA^∧^Asn stability and rescue mitochondrial dysfunction caused by mt-tRNA^∧^Asn variants (Lin et al., 2024). In addition, Q modification can protect cognate tRNAs, including tRNA^∧^His and tRNA^∧^Asn, against ribonuclease cleavage, suggesting a potential connection between Q-tRNA status, tRNA stability, and stress-induced tRNA fragment generation (Wang et al., 2018). A recent non-peer-reviewed preprint further suggested that mitochondrial stress may dynamically remodel multiple tRNA modifications, including Q-related modifications, and that QTRT1/QTRT2 deficiency may affect mitochondrial and metabolic pathways (Rashad et al., 2024). However, because this evidence remains preprint-level and was not generated in PD models, it should be interpreted as hypothesis-generating rather than definitive. Therefore, Q-tRNA modification should be described as a candidate interface linking microbial/nutritional input, translational quality control, and mitochondrial stress adaptation, rather than as an established master regulator of mitochondrial stress in PD.

### 3.2 tRNA modification and mitochondrial translational stress: a candidate molecular entry point for PD

**Figure 1:**
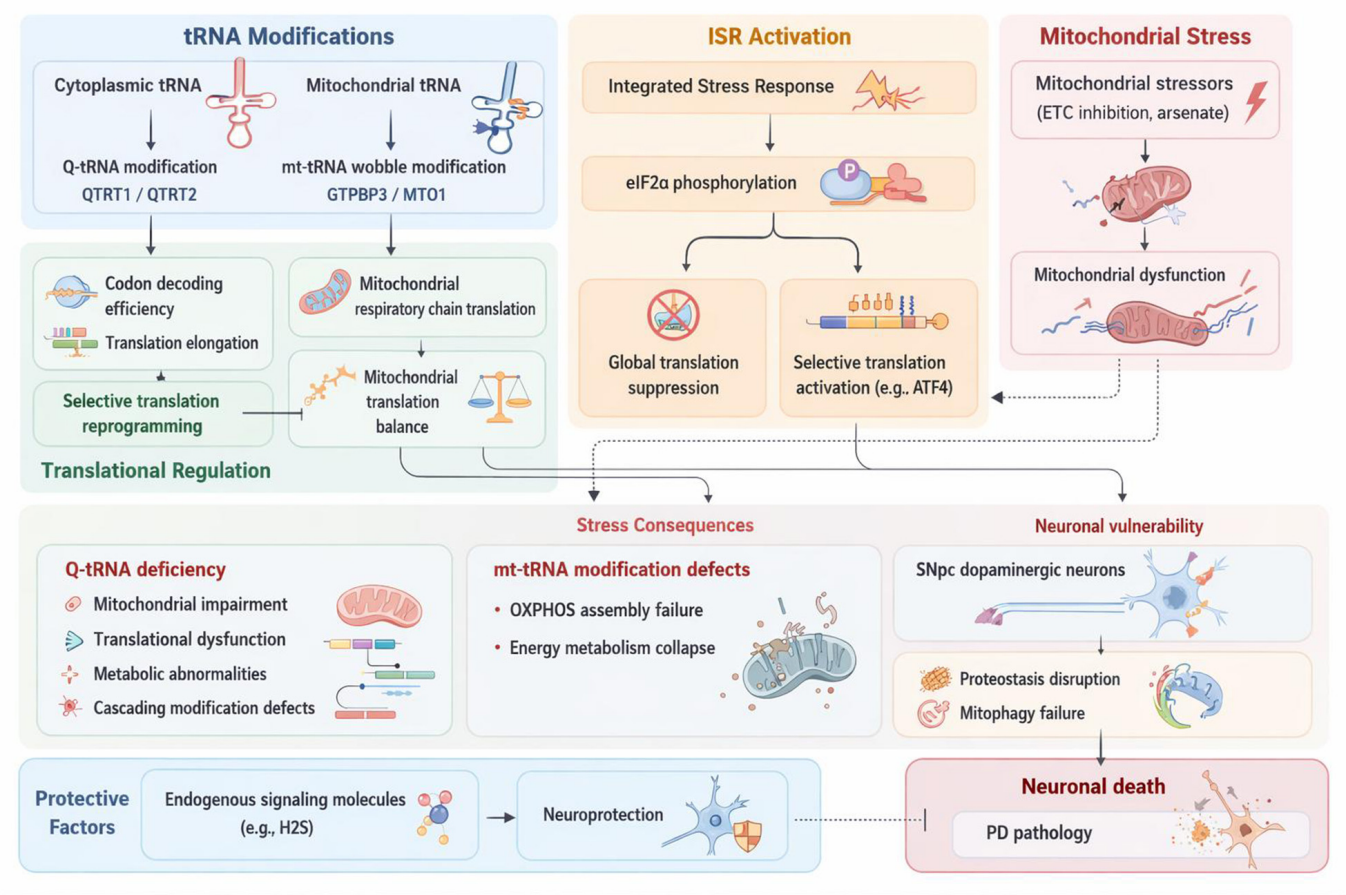
Evidence-stratified model linking tRNA modification, mitochondrial translational stress, and dopaminergic neuronal vulnerability in PD.

tRNA modifications constitute an important regulatory layer for codon decoding, translational fidelity, and stress-responsive protein synthesis. In mitochondrial stress states, these processes may become especially relevant because cells must coordinate reduced bioenergetic capacity with selective translation, proteostasis, and mitochondrial quality control. The following section discusses this relationship as a candidate mechanism relevant to PD vulnerability, while distinguishing peer-reviewed mitochondrial tRNA evidence from hypothesis-generating Q-tRNA evidence. Figure 1 presents an evidence-stratified schematic overview of the proposed relationship among tRNA modifications, mitochondrial translational stress, and dopaminergic neuronal vulnerability in PD.

This figure presents an evidence-stratified conceptual model rather than a fully established causal pathway. Solid arrows indicate mechanisms supported by peer-reviewed experimental evidence, whereas dashed arrows indicate proposed or incompletely validated links. Mitochondrial stressors, including electron transport chain impairment and oxidative stress, can activate the integrated stress response (ISR), characterized by eIF2α phosphorylation, global translational attenuation, and selective translation of stress-responsive transcripts such as ATF4. Peer-reviewed evidence supports the importance of mitochondrial tRNA wobble modifications, including τm^∧^5U-related pathways involving GTPBP3/MTO1, in maintaining respiratory-chain translation and oxidative phosphorylation. In contrast, the proposed role of cytoplasmic Q-tRNA modification in mitochondrial stress adaptation remains emerging and partly hypothesis-generating. QTRT1/QTRT2-mediated Q-tRNA modification may influence codon decoding and proteostasis, but direct evidence in PD models or SNpc dopaminergic neurons is currently lacking. Because SNpc dopaminergic neurons exhibit high bioenergetic demand, extensive axonal arborization, calcium-dependent pacemaking, and oxidative burden, failure of translational buffering may contribute to their selective vulnerability. Question marks in the figure denote untested links that require direct validation, including Q-tRNA profiling in PD tissue, causal manipulation of queuine/QTRT pathways, and exercise-intervention studies measuring tRNA modification outcomes.

#### 3.2.1 Why mitochondrial stress responses may intersect with tRNA modification

Mitochondrial dysfunction does not merely reduce ATP production; it can also reshape cellular translation, metabolism, and quality-control networks. In particular, the integrated stress response (ISR) is a major adaptive response to mitochondrial stress and is characterized by eIF2α phosphorylation, global attenuation of protein synthesis, and selective translation of stress-responsive programs such as ATF4 (Mick et al., 2020; Chakrabarty et al., 2024). Studies suggest that distinct mitochondrial defects can activate the ISR through different upstream mechanisms; for example, metabolic imbalance induced by electron transport chain inhibition can activate ISR signaling through GCN2, whereas other forms of mitochondrial damage may engage HRI-linked pathways related to mitochondrial quality control and mitophagy (Mick et al., 2020; Chakrabarty et al., 2024). This indicates that mitochondrial stress involves multiple translation reprogramming events that depend on the metabolic state and cellular environment.

tRNA modifications may intersect with the ISR because they influence codon-decoding efficiency, elongation kinetics, reading-frame fidelity, ribosomal pausing, and transcript-specific translational output (Nedialkova and Leidel, 2015; Agris et al., 2017). Importantly, the ISR is not merely a global shutdown of protein synthesis. Through phosphorylation of eIF2α, the ISR reduces general translation initiation while selectively enhancing the translation of stress-adaptive transcripts, particularly those regulated by upstream open reading frames, such as ATF4 (Pakos-Zebrucka et al., 2016; Young and Wek, 2016). Anticodon-loop and adjacent tRNA modifications can influence this selective translation process by altering codon optimality, ribosomal pausing, and decoding fidelity, thereby shaping which transcripts are efficiently translated under stress (Nedialkova and Leidel, 2015; Agris et al., 2017). Because mitochondrial dysfunction can activate the ISR in a defect- and metabolic-state-dependent manner, disruption of tRNA modification networks may reduce the capacity of cells to maintain adaptive translation under mitochondrial stress (Mick et al., 2020). In PD, however, this remains a plausible mechanism rather than a proven pathway, because direct evidence linking Q-tRNA modification defects to dopaminergic neuronal degeneration is still lacking.

#### 3.2.2 Q-tRNA as a candidate modulator of mitochondrial stress-linked translation

The relationship between Q-tRNA modification and mitochondrial stress should be interpreted cautiously. A recent non-peer-reviewed preprint reported that mitochondrial stress induced by electron transport chain inhibition or arsenite exposure was accompanied by dynamic remodeling of multiple tRNA modifications, including Q-related modifications, and that QTRT1 or QTRT2 loss affected mitochondrial and metabolic pathways (Rashad et al., 2024). This study is important because it provides hypothesis-generating evidence that Q-tRNA may participate in stress-responsive translational programs. However, because the study remains non-peer-reviewed and was not performed in PD models, it should not be used as definitive evidence that Q-tRNA is a “master regulator” of mitochondrial stress, particularly in PD.

Peer-reviewed studies provide more secure but still indirect support for a connection between queuine biology, translational quality control, and mitochondrial function. Tuorto et al. showed that nutritionally determined Q-tRNA levels promote Dnmt2-mediated methylation of tRNA^∧^Asp and control the translational speed of Q-decoded codons, thereby linking queuine availability to codon-specific translational regulation and proteostasis (Tuorto et al., 2018). Importantly, Q biology is not restricted to cytoplasmic tRNAs. Suzuki et al. identified Q34 in human mitochondrial tRNAs for Asp, His, Asn, and Tyr and showed that QTRT1 and QTRT2 are required for Q34 formation in these mitochondrial tRNAs, providing a direct molecular basis for linking QTRT-dependent queuosine biology to mitochondrial translation (Suzuki et al., 2020). In addition, Lin et al. reported that queuine supplementation improved mt-tRNA^∧^Asn stability and ameliorated mitochondrial dysfunction caused by mt-tRNA^∧^sAsn variants, further supporting a functional connection between queuine availability and mitochondrial homeostasis in a mitochondrial disease context (Lin et al., 2024). Moreover, Q modification can protect cognate tRNAs, including tRNA^∧^His and tRNA^∧^Asn, against ribonuclease cleavage, suggesting that altered Q-tRNA status may influence tRNA stability and stress-induced tRNA fragment generation (Wang et al., 2018).

Together, these findings justify considering Q-tRNA modification as a candidate interface between nutritional/microbial input, translational quality control, tRNA stability, and mitochondrial stress adaptation. Nevertheless, direct evidence showing altered Q-tRNA modification in PD brain tissue, PD biofluids, SNpc dopaminergic neurons, or exercise-treated PD models remains absent. Therefore, throughout this review, Q-tRNA should be described as a potential regulatory node rather than an established driver of mitochondrial stress in PD.

#### 3.2.3 mt-tRNA wobble modification and respiratory chain translational imbalance

Beyond cytoplasmic Q-tRNA biology, mitochondrial mt-tRNA wobble modifications are important for maintaining respiratory-chain protein synthesis and oxidative phosphorylation (OXPHOS). GTPBP3 and MTO1 collectively promote the τm5U modification at position 34 of mitochondrial tRNAs; abnormalities in this mechanism cause hypo-modified mt-tRNAs, mitochondrial translation disorders, and multisystem disease phenotypes (Peng et al., 2021). Research by Peng et al. clearly shows that GTPBP3 and MTO1 work together to enhance mt-tRNA wobble site modification, and mutations linked to these proteins disrupt the modification process, leading to disease (Peng et al., 2021). Such evidence indicates that abnormalities in mitochondrial tRNA modification can directly impair respiratory-chain translation and compromise energy metabolism in specific genetic and experimental contexts. Classic studies have shown that deficiencies in wobble-site modifications can directly affect codon-anticodon pairing and impair mitochondrial translation. In a MERRF-related mt-tRNA^∧^Lys mutant model, Yasukawa et al. reported that loss of wobble modification markedly reduced translational activity for the cognate codon and was associated with reduced oxygen consumption and mitochondrial dysfunction (Yasukawa et al., 2001). Therefore, mt-tRNA wobble modifications are functionally important for accurate respiratory-chain translation and OXPHOS maintenance. Integrating these findings with the Q-tRNA literature suggests that cytoplasmic and mitochondrial anticodon-associated tRNA modifications may share a broader role in supporting translational accuracy and metabolic continuity under stress conditions.

#### 3.2.4 From translational stress to selective vulnerability of nigral neurons

A major pathological characteristic of PD is the targeted degeneration of dopaminergic neurons in the substantia nigra pars compacta (SNpc). Accumulating evidence indicates that this vulnerability is closely linked to their high energy demand, oxidative burden, and sustained mitochondrial load (Ni and Ernst, 2022; Watanabe et al., 2024). According to Ni and Ernst, SNpc dopaminergic neurons have elevated basal metabolic rates and ATP needs, which makes them particularly vulnerable to OXPHOS damage and complex I deficiency (Ni and Ernst, 2022); Watanabe et al. further emphasize that these neurons also endure high dopamine turnover, calcium oscillations, and the resulting oxidative pressure, subjecting them to lifelong bioenergetic challenges (Watanabe et al., 2024). In this context, tRNA modification networks may become particularly relevant. SNpc neurons, characterized by autonomous activity, extensive axonal arborization, and high mitochondrial dependence, may require robust codon-decoding fidelity and translational buffering capacity to maintain proteostasis, mitochondrial turnover, and stress adaptation. When Q-tRNA or mt-tRNA wobble modifications are impaired, cells may become more vulnerable to translational imbalance under stress, potentially contributing to mitochondrial dysfunction, proteostatic burden, and neuronal injury. Evidence from related PD models suggests that maintaining mitochondrial homeostasis and limiting oxidative stress can improve the stress tolerance of nigral dopaminergic neurons. Hydrogen sulfide, an endogenous gas signaling molecule, has shown distinct neuroprotective effects in Parkinson's disease through this mechanism (Ye et al., 2024). Hence, Q-tRNA deficiency should be considered a testable candidate contributor to translational-buffering failure rather than a confirmed event in PD. This candidate mechanism offers a useful entry point for future studies linking RNA modification, mitochondrial stress, and selective neuronal vulnerability, but it requires direct validation in SNpc dopaminergic neurons, PD animal models, and patient-derived samples.

### 3.3 Aberrations in tRNA biology in PD: from tRFs to pathological propagation

**Figure 2:**
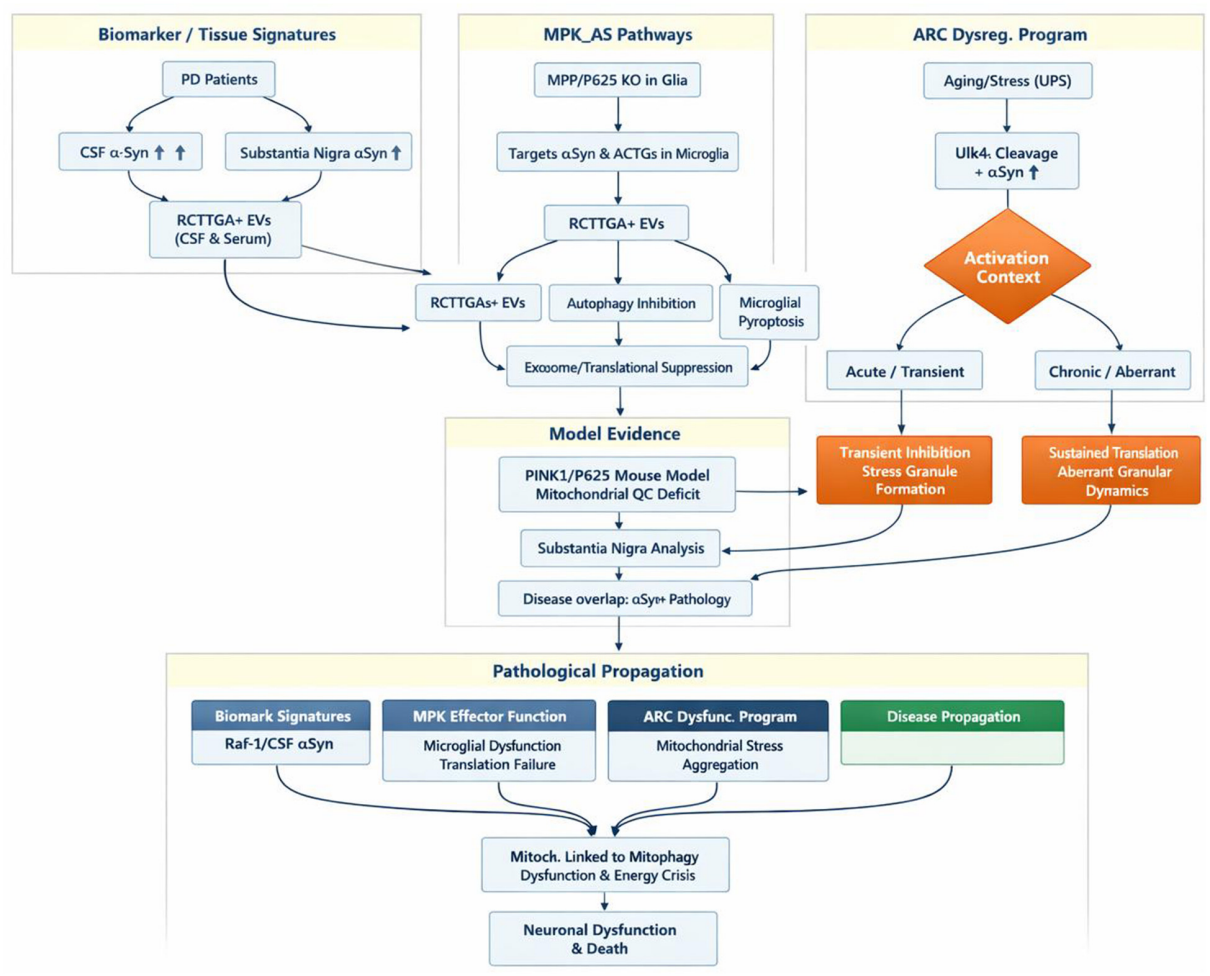
tRNA fragments (tRFs) in PD: from biofluid biomarkers to candidate pathological effectors.

Building upon the role of tRNA modifications in mitochondrial translational stress, the next section expands the discussion to another layer of tRNA biology-tRNA-derived fragments (tRFs). Emerging evidence suggests that tRFs may serve not only as candidate fluid biomarkers for PD but also as potential contributors to neuroinflammation, translational reprogramming, and disease propagation. Figure 2 shows tRNA fragments (tRFs) in PD: from biofluid biomarkers to candidate pathological effectors.

This figure summarizes the multifaceted roles of tRNA-derived fragments (tRFs) in PD pathology. Left panel (Biofluid and Tissue Signatures): PD patients exhibit disease-specific tRF profiles in cerebrospinal fluid (CSF), blood, and substantia nigra. A conserved RGTTCRA motif-containing tRF is elevated, while mitochondria-derived tRFs (MT-tRFs) are decreased; this ratio shift is detectable even in pre-symptomatic individuals. Middle panel (tRFs as Effector Molecules): PD-associated tRFs are not merely biomarkers. (1) RGTTCRA-tRFs may exacerbate translational suppression via complementary pairing with ribosomal RNA. (2) tRF-02514, carried by extracellular vesicles (EVs), enters microglia, targets ATG5, inhibits autophagy, and triggers pyroptosis, releasing inflammatory factors that promote neuronal loss. Right panel (ANG/tRNA Biphasic Program): Under acute stress, angiogenin (ANG) cleaves tRNAs to generate tiRNAs, which inhibit translation and promote stress granule formation—a protective response. However, chronic or aberrant ANG activation in PD may cause sustained translational suppression, altered RNA granule dynamics, and pathological consolidation. Bottom panel (Model Evidence): In parkin/POLG mice, a mitochondrial quality-control model, disease-associated tsRNA signatures have been observed in the substantia nigra, supporting the possibility that tRNA biology abnormalities occur in PD-relevant brain regions rather than only in peripheral biofluids. Together, these findings support tRFs as candidate diagnostic indicators and potential contributors to PD-related translational stress and inflammatory propagation, although their causal roles require further validation.

#### 3.3.1 Abnormalities in tRNA fragments in biofluids and brain tissue of PD patients

Increasing evidence indicates that PD is associated not only with protein aggregation issues, mitochondrial damage, or inflammation but also with noticeable and disease-specific changes in the small RNA landscape, particularly tRFs. Paldor et al., assessing small RNA sequencing data from cerebrospinal fluid (CSF) and blood, reported that PD patients exhibited sets of tRFs in both CSF and blood that could discriminate patients from controls; Moreover, the tRF composition in CSF was considerably different from that in blood, indicating that these molecules are not merely peripheral “background noise,” but may reflect, to some extent, the state of the CNS (Paldor et al., 2023). This finding is significant because it extends the analysis of tRNA biology abnormalities in PD from postmortem or tissue-based samples to more accessible biofluids.

This direction was further supported by a 2025 study. Madrer et al. reported that PD-associated tRFs carrying a conserved sequence motif (RGTTCRA-tRFs) were increased in the substantia nigra, CSF, and blood of PD patients, whereas mitochondria-derived tRFs (MT-tRFs) showed a relative decrease. More notably, the study also reported that a detection scheme according to increased RGTTCRA-tRFs and reduced MT-tRFs in whole blood could discriminate pre-symptomatic individuals from controls, and asymptomatic carriers of PD-associated mutations also exhibited a higher RGTTCRA-tRFs/MT-tRFs ratio (Madrer et al., 2025). This means that tRF abnormalities in PD are not restricted to the end-stage of the disease, but may appear before the full manifestation of clinical motor symptoms, conferring tRFs with the potential to enter frameworks for “prodromal stratification” and “early identification.”

#### 3.3.2 tRFs are not just biomarkers, but also potential effector molecules

The understanding of tRFs/tiRNAs has evolved substantially beyond the earlier view that they are merely tRNA degradation byproducts. Classic studies have shown that angiogenin (ANG) can cleave tRNAs under stress conditions, generating tiRNAs that mediate translational suppression independently of the canonical eIF2α pathway; further evidence reported that notable 5′-tiRNAs can facilitate stress granule assembly, and short fragments obtained from the 5′ end of tRNAs can directly inhibit protein translation in human cells (Yamasaki et al., 2009; Emara et al., 2010; Sobala and Hutvagner, 2013). Therefore, tRFs/tiRNAs have at least three functions relevant to neurodegenerative contexts: (1) modulation of translation initiation and elongation; (2) participation in stress-granule formation and RNA-protein complex remodeling; and (3) rapid regulation of cellular stress and survival responses. Specifically, the tRF abnormalities identified in PD are not merely passive indicators, but might be involved in disease-related translational reprogramming.

In the context of PD, the dual function of tRFs as both readouts and regulators is beginning to emerge. First, the Nature Aging study not only reported the elevation of PD-specific tRF motifs in the substantia nigra, CSF, and blood, but also revealed that these RGTTCRA-tRFs may exacerbate translational suppression via complementary pairing with ribosomal RNA and imbalance with the translation-supporting LeuCAG3-tRF (Madrer et al., 2025). Second, Dong et al. reported in 2025 that tRF-02514 in peripheral extracellular vesicles (EVs) could promote microglial pyroptosis, inflammatory factor release, and neuronal loss by targeting ATG5 and inhibiting autophagy; In contrast, inhibiting this tRF alleviated these pathological changes and delayed PD progression (Dong et al., 2025). This discovery is important because it suggests that at least some PD-associated tRFs may act beyond correlative biomarkers and function as candidate effector molecules in microglia-neuron pathological communication. However, this conclusion remains tRF-specific and model-dependent; broader validation across independent PD cohorts, cell types, and disease stages is still needed.

#### 3.3.3 tsRNA signatures in PD models

Research using animal models further reinforces the idea that “abnormalities in tRNA biology are not merely peripheral occurrences.” Baindoor et al., performing small RNA sequencing analysis on different neurodegenerative disease models, reported that different disease models have distinguishable tsRNA signatures in their respective disease-affected brain regions; specifically, in the PD model utilizing substantia nigra samples from parkin/POLG mice, the researchers determined a disease-specific tRF composition profile (Baindoor et al., 2024). This indicates that in a PD model with underlying genetic mitochondrial stress, tsRNA abnormalities have affected the crucial brain region associated with the disease, rather than being confined to blood or other peripheral tissues. The importance of this finding lies not only in “tRF changes also occur in models,” but also in further linking tsRNAs with classic PD mechanisms—especially mitochondrial dysfunction. The parkin/POLG model itself represents a background of impaired mitochondrial quality control and mitochondrial DNA homeostasis, and the substantia nigra is one of the most vulnerable regions in PD; observing disease-specific tsRNA profiles under these conditions reveals that tsRNA modifications are similarly coupled with neuronal energy crisis, translational burden, and adaptive failure (Baindoor et al., 2024). Therefore, abnormalities in tRNA biology in PD should be considered a disease-relevant molecular layer rather than merely peripheral epiphenomena. However, whether these abnormalities act as causal drivers, adaptive responses, or downstream markers remains to be clarified.

#### 3.3.4 The biphasic nature of the ANG/tRNA cleavage stress program in PD

ANG-mediated tRNA cleavage appears to show a context-dependent biphasic pattern. Under acute stress, ANG-generated tiRNAs can rapidly suppress translation, promote stress-granule formation, and help cells shift from high energy expenditure toward resource conservation and repair (Yamasaki et al., 2009; Emara et al., 2010). In this context, the ANG-tRNA cleavage program may act as a short-term adaptive mechanism that helps cells withstand oxidative, heat, and metabolic stress. On the other hand, if this program is chronically, aberrantly, or maladaptively activated, the consequences may shift from “adaptation” to a “consolidated pathological state.” Prehn and Jirström summarized evidence that ANG variants have been identified in PD and that exogenous ANG has shown neuroprotective effects in several neurodegeneration models. At the same time, ANG-induced tRNA fragments are tightly linked to translation suppression, suppression of ribosome biogenesis, and regulation of anti-apoptotic pathways (Prehn and Jirström, 2020). This suggests that, in PD, the ANG/tRNA cleavage axis is not simply “beneficial” or “harmful”; rather, its effects may depend on the timing, intensity, and duration of activation: transient activation may help neurons withstand acute stress, whereas chronic activation could reinforce translational suppression, sustain aberrant RNA-granule dynamics, and contribute to inflammation and neuronal dysfunction. Therefore, this part serves as a natural link to the upcoming discussion on how “protective adaptation can evolve into pathological consolidation.”

A key unresolved question is what determines the switch from adaptive ANG-mediated tRNA cleavage to maladaptive chronic tRF accumulation. Current evidence suggests that this switch is unlikely to be controlled by a single molecule. Instead, it may depend on the convergence of stress duration, ANG subcellular localization, ribonuclease activity, oxidative state, ribonuclease inhibitor availability, stress-granule clearance capacity, and the balance between transient translational repression and persistent proteostatic collapse. ANG was originally identified as a stress-activated ribonuclease that cleaves mature tRNAs under oxidative, heat-shock, and ultraviolet stress conditions, generating tiRNAs that contribute to translational repression (Yamasaki et al., 2009). Under acute stress, ANG-generated tiRNAs can suppress translation initiation and promote stress granule assembly, thereby reducing energy expenditure and supporting short-term survival (Emara et al., 2010; Ivanov et al., 2011). The activity and localization of ANG are further regulated by ribonuclease/angiogenin inhibitor 1 (RNH1), which controls stress-induced ANG relocalization and thereby influences its growth- and survival-related functions (Pizzo et al., 2013). In chronic PD-like stress, persistent mitochondrial dysfunction, oxidative stress, α-synuclein proteostatic burden, and impaired autophagic clearance may convert this transient adaptive response into sustained translational repression, abnormal RNA granule persistence, and inflammatory amplification (Lashuel et al., 2013; Schapira, 2007; Prehn and Jirström, 2020; Wong and Krainc, 2017; Bose and Beal, 2016; Pakos-Zebrucka et al., 2016). This interpretation is consistent with the broader view that ANG and tRNA fragments are implicated in PD and neurodegeneration, but that their effects are context-dependent rather than uniformly protective or pathogenic (Prehn and Jirström, 2020). Because defective autophagic clearance of stress granules has been proposed as one route through which stress granules and associated protein aggregates become pathological, impaired stress-granule turnover may represent one mechanism linking chronic tRF accumulation to neurodegenerative proteostasis failure (Ryan and Rubinsztein, 2024). Thus, the “molecular switch” is better conceptualized as a threshold-dependent transition from reversible stress adaptation to chronic RNA-proteostasis failure rather than as a single binary regulator.

### 3.4 How exercise engages this axis: from peripheral metabolism to circulating RNA carriers

**Figure 3:**
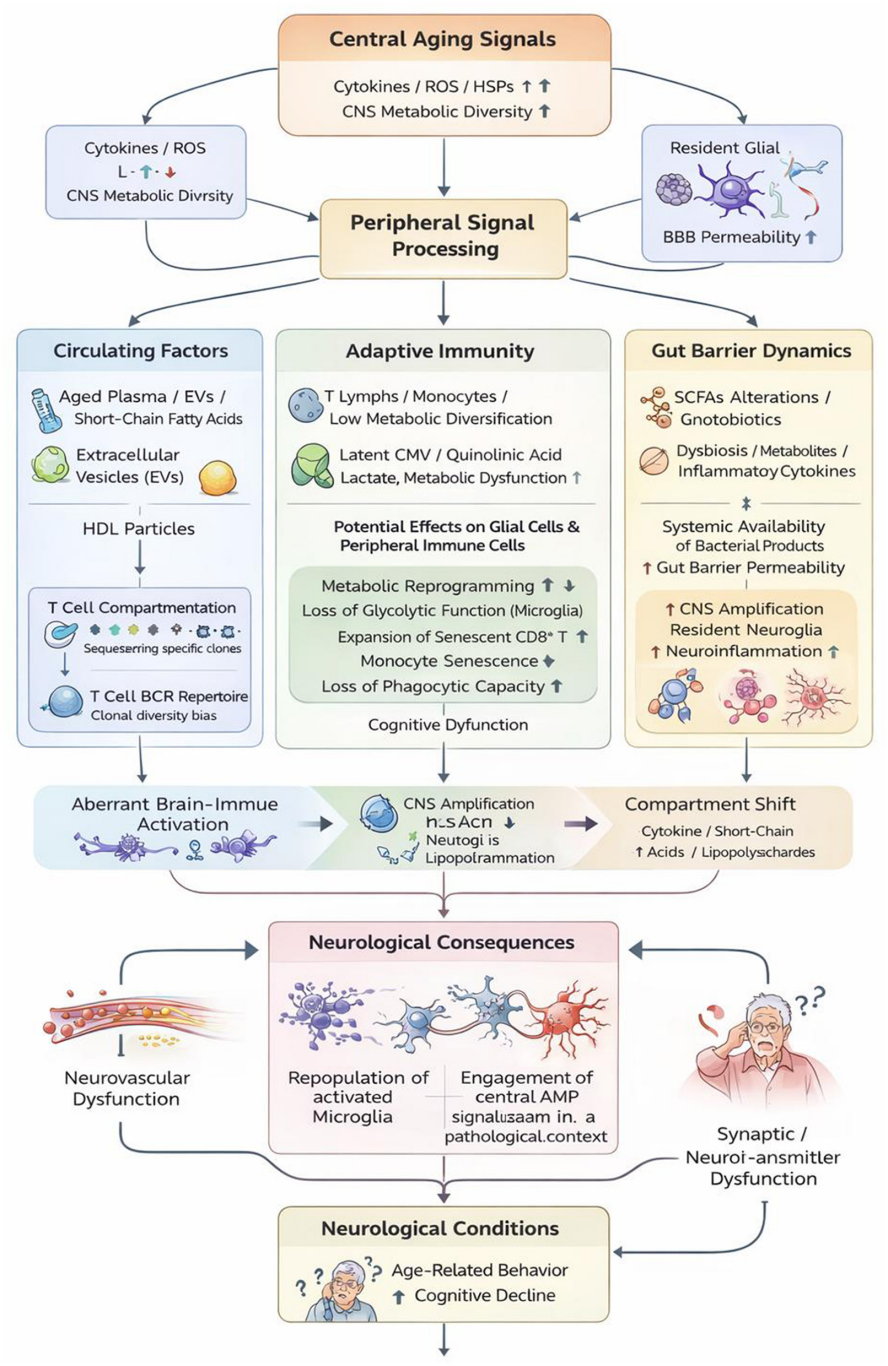
Exercise may engage the tRNA modification and tRF axis in PD: from peripheral metabolism to circulating RNA carriers.

Having discussed tRFs as candidate biomarkers and potential effector molecules in PD-related pathology, the question arises: can external interventions modulate this tRNA-centered axis? Exercise, a promising non-pharmacological strategy, may reshape peripheral metabolism and circulating RNA signaling, thereby potentially engaging tRNA-related regulatory networks through multiple interconnected pathways. Figure 3 illustrates the proposed ways in which exercise may engage the tRNA modification and tRF axis in PD, from peripheral metabolism to circulating RNA carriers.

This figure summarizes the proposed mechanisms by which exercise may modulate tRNA-related biology in the context of PD. Top panel (Exercise Intervention): Clinical and preclinical studies suggest that moderate-intensity aerobic exercise can improve PD-related outcomes and may engage neuroprotective mechanisms, accompanied by remodeling of gut microbiota, metabolic profiles, and circulating exosomal RNA cargo. Left panel (Circulating RNA Carriers): Acute exercise dynamically alters the small RNA composition of circulating carriers—extracellular vesicles (EVs) and high-density lipoproteins (HDL)—in a carrier-specific manner. EVs show increased miRNA abundance, while HDL particles exhibit increased relative abundance of tRNA-derived small RNAs (tDRs/tRFs). These exercise-responsive tRFs may function as candidate relay molecules linking peripheral tissues, such as muscle, liver, and immune cells, to the brain. Right panel (Metabolic Remodeling): Exercise intensity-dependently elevates metabolites such as lactate, succinate, and TCA cycle intermediates. Notably, aminoacyl-tRNA synthetases (AARS1/AARS2) function as intracellular lactate sensors, participating in lactylation reactions (Li et al., 2024). This raises the hypothesis that exercise-induced metabolic shifts could modulate tRNA-modifying enzyme activity, substrate availability, or translation complex assembly—though direct evidence in PD remains to be established. Bottom panel (Gut Microbiota-Queuine Axis): Exercise has been reported to remodel PD-associated gut microbiota composition, including taxa such as Clostridia and Roseburia, in association with symptom improvement. Since queuine—the precursor for Q-tRNA modification—is exclusively sourced from gut microbiota and diet, exercise-induced microbial shifts may influence host queuine availability (Ehrenhofer-Murray, 2025; Tuorto et al., 2018; Rashad, 2025; Dong et al., 2025). Recent evidence that the microbial metabolite preQ1 can compete with queuine for tRNA modification sites further supports the plausibility of this gut-translation connection (Zhang et al., 2025). Collectively, these pathways support the hypothesis that exercise may act as an upstream peripheral signal remodeler capable of engaging the tRNA modification and tRF network, offering a testable framework for understanding its potential disease-modifying effects in PD.

#### 3.4.1 Exercise as a promising non-pharmacological intervention in PD

The value of exercise in PD should not be viewed only as functional training or rehabilitation support; it may also act as an upstream intervention that reshapes peripheral metabolism, immunity, and circulating signals (Ahlskog, 2018; Petzinger et al., 2013; Pizzo et al., 2013; Wei et al., 2025; Karvinen et al., 2023; Denham and Spencer, 2020; Allen et al., 2018; Mailing et al., 2019). Clinically, Wei et al. reported that an 8-week moderate-intensity aerobic exercise program in patients with PD reduced MDS-UPDRS III scores and was associated with changes in gut microbiota composition, bile acid metabolism, and inflammatory cytokine profiles (Wei et al., 2025). This suggests that exercise in PD may influence symptomatic, metabolic, and inflammatory domains, rather than only improving muscle strength or walking ability. This upstream regulatory interpretation is also supported by preclinical evidence. Citron et al. reported in an α-syn PFF rat model that 1 month of moderate-intensity treadmill exercise slowed the loss of dopaminergic neurons in the substantia nigra pars compacta, along with modifications in the RNA cargo of circulating plasma exosomes (Citron et al., 2025). This suggests that exercise may influence brain pathology partly through circulating peripheral signaling molecules. Together with the tRF/EV literature, these findings support the possibility that exercise may remodel peripheral-to-central RNA signaling rather than acting solely as a symptomatic intervention.

#### 3.4.2 Exercise modifies the circulating small RNA ecosystem: tRNA-derived sRNAs in EVs and HDL

Recent findings indicate that short-term physical activity can alter the composition of small RNAs in circulating carriers (Oliveira et al., 2018; Karvinen et al., 2023; Denham and Spencer, 2020). In a rat study, Oliveira Jr. et al. reported that a single bout of acute aerobic exercise not only increased serum EV concentration, but also altered the small RNA content carried by EVs; in their sequencing results, besides miRNAs, modifications in tRNA molecules linked to exercise were detected, with one tRNA being downregulated post-exercise (Oliveira et al., 2018). Although this study did not fully evaluate the physiological roles of tRFs, it showed that EVs involved in post-exercise humoral communication carry RNA species beyond miRNAs, including exercise-responsive tRNA-related molecules. More detailed human studies further indicate that different circulating carriers respond differently to exercise. Karvinen et al. reported that after acute exercise, the sRNA composition of EVs and HDL particles changed in divergent directions: miRNAs were comparatively increased in EVs, while an increased relative abundance of transfer RNA-derived small RNAs (tDRs) was uncovered in HDL particles (Karvinen et al., 2023). This suggests that exercise may not only alter the abundance of RNA signals but also redistribute RNA species among different carriers, thereby influencing signal stability, tissue distribution, and potential recipient-cell interactions. Combined with preclinical PD evidence that moderate-intensity exercise can modify plasma exosomal RNA in association with neuroprotective outcomes (Citron et al., 2025), it is plausible to hypothesize that exercise-related tRFs/tDRs may serve as candidate relay molecules connecting skeletal muscle, liver, immune system, and the brain.

A further mechanistic issue is how exercise-responsive EV- or HDL-associated RNAs could communicate with the CNS. Current evidence indicates that peripheral EVs can cross the blood-brain barrier (BBB), but this transport is selective, context-dependent, and influenced by inflammation, endothelial uptake, vesicle surface proteins, and recipient-cell state. Experimental work has shown that EVs can undergo BBB transport and that inflammatory conditions can alter their brain pharmacokinetics (Banks et al., 2020). Therefore, exercise-modified EV RNA cargo should not be assumed to freely access the brain; rather, its CNS relevance requires direct testing using labeled EV tracking, endothelial transcytosis assays, brain-region-specific uptake mapping, and recipient-cell RNA profiling. HDL-associated small RNAs represent an additional route of peripheral RNA transport. HDL particles can carry small RNAs and deliver them to recipient cells, and exercise has been reported to increase the relative abundance of tRNA-derived small RNAs in HDL particles (Karvinen et al., 2023). However, whether HDL-associated tRFs directly cross the BBB, transfer RNA cargo to brain endothelial cells, or indirectly modulate CNS function through vascular and immune signaling remains unresolved. The manuscript therefore treats EV/HDL-associated tRFs as candidate peripheral-to-central signaling molecules rather than established brain-delivered effectors.

#### 3.4.3 Exercise-induced metabolic remodeling may influence tRNA modifying enzyme activity and substrate supply

At present, there is no direct evidence that exercise alters Q-tRNA modification levels in PD brain tissue, peripheral blood, or disease-relevant cellular models. The proposed connection between exercise-induced metabolic remodeling and tRNA-modifying enzyme activity should therefore be treated as a testable hypothesis rather than an established mechanism. Randomized crossover trials have shown that exercise intensity strongly influences the magnitude of changes in circulating metabolites; after strenuous exercise, lactate, succinate, Lac-Phe, TCA-cycle intermediates, and metabolites related to amino acid and acetylation pathways showed marked intensity-dependent increases (Weber et al., 2025). These changes indicate that after exercise, the internal environment is dynamic, quickly transitioning to a state characterized by elevated lactate levels, improved energy conversion, and alterations in amino acid metabolism (Weber et al., 2025). This is pertinent to tRNA biology as numerous tRNA-associated proteins possess the ability to sense metabolic changes. In 2024, two separate studies demonstrated that AARS1 and AARS2 function as intracellular sensors for L-lactate, directly interacting with lactate and engaging in lactylation-related processes (Li et al., 2024). Although this does not demonstrate that exercise modifies tRNA modifications through AARS1/2, it indicates that aminoacyl-tRNA synthetases, a protein family closely linked to translation, can directly sense lactate, one of the classic metabolic signals of exercise. By extension, exercise-induced changes in lactate, redox state, amino acid flux, and methyl/acetyl donor availability could plausibly influence the RNA modification landscape by affecting modifying-enzyme activity, substrate availability, or translation-complex assembly. In the context of PD, this should be viewed as a plausible molecular hypothesis requiring experimental validation rather than as a proven mechanism.

#### 3.4.4 Exercise may influence queuine supply through the gut microbiota

The gut microbiota represents one plausible link between exercise and the queuine-Q-tRNA axis. Existing research indicates that changes in gut microbiota composition occur in PD during both prodromal and clinical phases and may be linked to symptoms and disease progression (Peters et al., 2025). Human and animal studies further suggest that aerobic exercise can remodel the PD-associated gut microbiota: in humans, an 8-week aerobic exercise intervention was associated with changes in Clostridia, Roseburia, microbial diversity, bile acid metabolism, and inflammatory factors in patients with PD (Wei et al., 2025); In mice, exercise improved PD-related cognitive and pathological outcomes, but these protective benefits were significantly reduced when antibiotics depleted the microbiota (Shan et al., 2025). For instance, in a mouse model of chronic REM sleep deprivation, ozone rectal insufflation ameliorated cognitive dysfunction while at the same time regulating gut microbiota and inflammatory responses, supporting the wider concept that peripheral microbiota-targeted interventions can impact brain outcomes through gut-brain signaling (Cheng et al., 2024). These findings collectively suggest that some beneficial effects of exercise may depend partly on microbiota-linked peripheral signaling. This aligns naturally with the queuine axis, as queuine itself is not synthesized by the eukaryotic host, but is mainly obtained from the gut microbiota and diet; recent reviews also explicitly state that Q modification is tightly linked to oxidative stress, mitochondrial stress, and nervous system health (Rashad, 2025). Furthermore, a study from 2025 reported that the microbial metabolite preQ1 can compete with queuine for modification sites on host tRNA, thereby affecting cell growth and translation quality control (Zhang et al., 2025). From a mechanistic perspective, it is plausible to consider a model in which exercise alters PD-related gut microbiota structure and metabolic output, thereby influencing host queuine availability and the potential for Q-tRNA modification. Admittedly, the entire causal chain linking exercise, microbiota, queuine, Q-tRNA and PD progression still requires systematic stepwise validation; nevertheless, it holds great potential to underpin the paper's central hypothesis as a key mechanistic link.

An additional layer of complexity is the competitive relationship between queuine and the microbial metabolite pre-queuosine 1 (preQ1). Recent evidence indicates that preQ1, a microbial intermediate in the bacterial queuosine biosynthetic pathway, can compete with queuine for modification of the same host tRNA sites and can influence mammalian cell proliferation and translation quality control (Zhang et al., 2025). This finding suggests that PD-associated dysbiosis may not simply reduce or increase total queuine availability; it could also alter the ratio between queuine, preQ1, and related microbial intermediates. Functionally, an unfavorable preQ1/queuine balance could modify the efficiency or quality of host tRNA modification, thereby affecting codon decoding, translational fidelity, and stress adaptation (Zhang et al., 2025). However, no study has yet measured queuine/preQ1 ratios in PD patients, PD animal models, or exercise interventions. Future work should combine metagenomics, targeted metabolomics, and Q-tRNA profiling to test whether exercise shifts microbial queuine/preQ1 metabolism in a direction that supports host translational homeostasis.

### 3.5 Evidence map of the proposed causal chain

The exercise-microbiota-queuine-tRNA modification axis should be viewed as a modular framework rather than a continuous proven pathway. The strongest evidence supports individual components of the model: microbial/dietary sources of queuine, QTRT1/QTRT2-mediated Q-tRNA modification, Q-tRNA effects on codon decoding and proteostasis, PD-associated tRF signatures, exercise-induced microbiota remodeling, and exercise-responsive changes in circulating RNA carriers (Paldor et al., 2023; Tuorto et al., 2018; Lin et al., 2024; Wei et al., 2025; Karvinen et al., 2023; Madrer et al., 2025; Suzuki et al., 2020). In contrast, the weakest links are those connecting these components into a single causal pathway in PD. Specifically, no study has yet demonstrated that exercise increases queuine availability in PD, that exercise modifies Q-tRNA levels in the SNpc or striatum, or that Q-tRNA remodeling mediates exercise-induced dopaminergic neuroprotection.

Accordingly, this review interprets the proposed axis as a sequence of testable modules: (1) exercise modifies systemic metabolism and gut microbiota (Wei et al., 2025); (2) microbiota and diet shape queuine/preQ1 availability (Tuorto et al., 2018; Zhang et al., 2025); (3) queuine availability may influence QTRT1/QTRT2-mediated Q-tRNA modification (Suzuki et al., 2020); (4) Q-tRNA modification may affect translational fidelity, ribosomal pausing, and proteostasis (Tuorto et al., 2018; Wang et al., 2018); (5) altered tRF production may reflect or amplify translational stress (Paldor et al., 2023; Madrer et al., 2025); (6) translational stress may interact with mitochondrial dysfunction and neuroinflammation (Bose and Beal, 2016; Mick et al., 2020; Pakos-Zebrucka et al., 2016); (7) these processes may influence SNpc dopaminergic neuronal vulnerability (Surmeier et al., 2017; Ni and Ernst, 2022; Watanabe et al., 2024).

Steps 1, 2, 4, and parts of 5 are supported by peer-reviewed evidence in relevant or adjacent systems. Steps 3, 6, and 7 remain incompletely validated in PD-specific models, and none has yet been linked experimentally into a continuous exercise-responsive causal chain. The full chain, especially the exercise → microbiota → queuine/preQ1 → Q-tRNA → dopaminergic neuroprotection sequence, remains an experimental priority.

### 3.6 A state-dependent conceptual model of the exercise-microbiota-queuine-tRNA axis in PD

**Figure 4:**
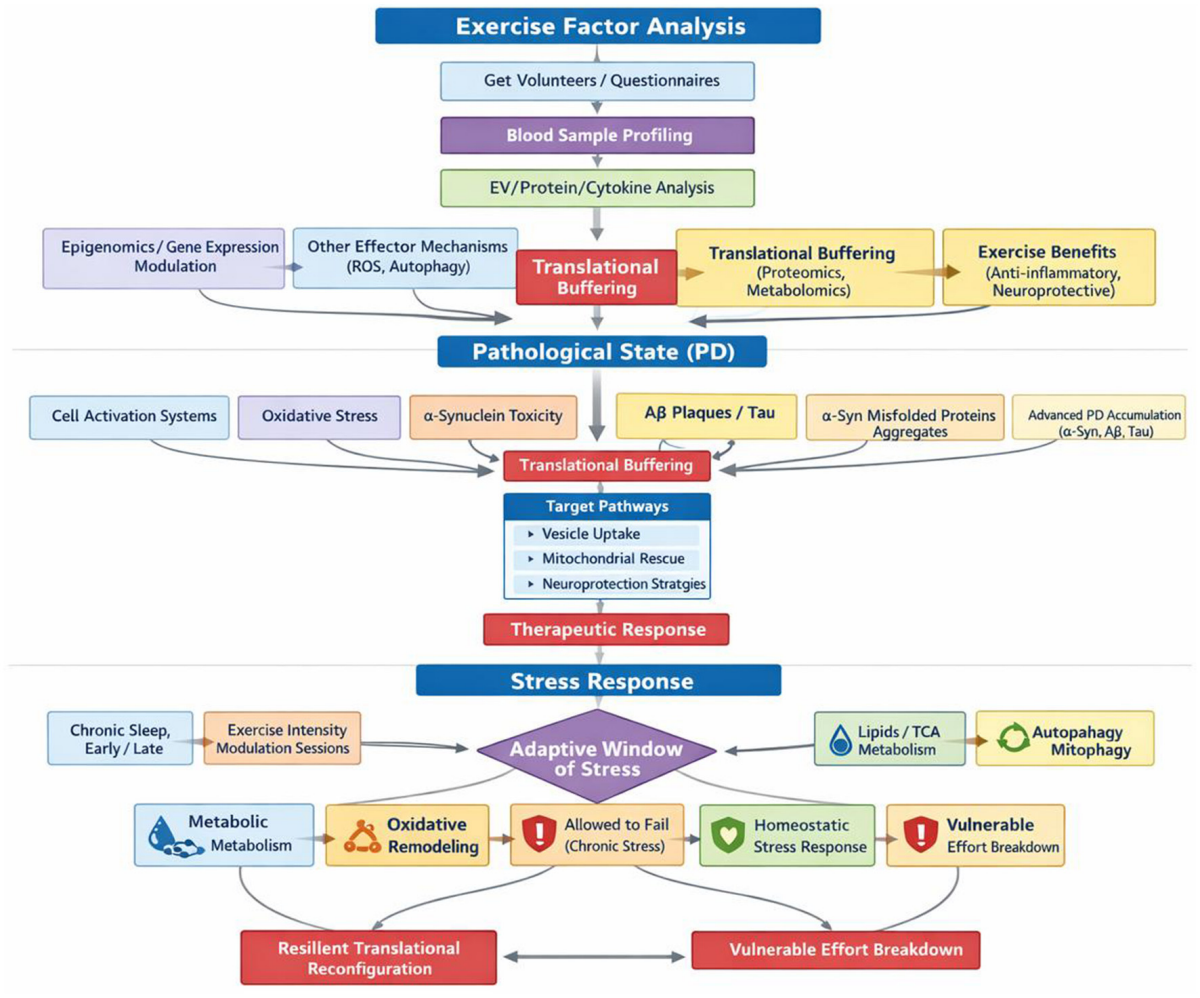
Evidence-stratified adaptive-window model of the proposed exercise-microbiota-queuine-tRNA modification axis in PD.

Synthesizing the evidence presented in preceding sections, we propose a state-dependent conceptual model in which exercise, gut microbiota, queuine metabolism, and tRNA modifications may converge on neuronal translational homeostasis. This model is intended to organize current evidence and uncertainties rather than to define a confirmed pathogenic pathway, and it considers how this network may influence PD vulnerability across baseline, pathological, and interventional states (Figure 4).

This figure illustrates a hypothesis-generating adaptive-window model rather than a confirmed causal pathway. Solid arrows represent links supported by peer-reviewed evidence; dashed arrows represent plausible but untested links; question marks indicate experimental gaps. In the baseline state, microbial/dietary queuine can be salvaged by host cells and incorporated into selected tRNAs by the QTRT1/QTRT2 complex, potentially supporting codon-decoding fidelity and proteostatic stability. In the pathological PD state, established mechanisms such as α-synuclein aggregation, mitochondrial dysfunction, oxidative stress, and neuroinflammation may impose sustained stress on translational homeostasis. Emerging evidence indicates that PD is associated with altered tRF profiles in blood, CSF, and substantia nigra, but direct evidence for Q-tRNA deficiency in PD remains lacking. In the interventional state, exercise may reshape systemic metabolism, circulating RNA carriers, and gut microbiota composition. These effects could, in principle, alter queuine/preQ1 availability, Q-tRNA modification, tRF generation, and mitochondrial translational stress; however, the complete exercise → microbiota → queuine/preQ1 → Q-tRNA → dopaminergic neuroprotection pathway has not yet been demonstrated. The adaptive-window concept proposes that exercise may be most beneficial when disease stage is early, exercise intensity is moderate, microbiota function is supportive, and translational-stress plasticity remains preserved. Excessive exercise, advanced disease, severe dysbiosis, or established translational collapse may limit or modify the expected benefit.

#### 3.6.1 Baseline state: the “translational buffering system” under healthy conditions

Under physiological conditions, protein synthesis is regulated by a coordinated network of mRNA abundance, codon usage, ribosomal kinetics, aminoacyl-tRNA availability, and tRNA modifications (Kirchner and Ignatova, 2015; Nedialkova and Leidel, 2015). Within this network, Q-tRNA modification may contribute to translational stability by fine-tuning codon decoding and elongation speed, although its quantitative contribution likely varies across tissues, metabolic states, and cell types. Specifically, tRNA modifications are seen as an essential regulatory layer for maintaining the accuracy of codon decoding, the efficiency of translation, and the homeostasis of protein folding (Kirchner and Ignatova, 2015; Nedialkova and Leidel, 2015; Agris et al., 2007). Over the past decade, evidence has progressively progressed the concept of a “translational buffering system,” whereby cells coordinately modulate codon recognition, translation speed, and translational fidelity via various tRNA modifications to sustain a stable protein synthesis program amidst metabolic fluctuations or environmental changes (Kirchner and Ignatova, 2015; Nedialkova and Leidel, 2015; Agris et al., 2007; Begley et al., 2007). Within this framework, tRNA modifications not only determine how the ribosome interprets the genetic code but also contribute to maintaining proteome stability by affecting ribosomal pauses, translation speed, and the protein folding environment.

Among the numerous tRNA modifications, queuosine (Q) modification has unique physiological origins and regulatory features (Ehrenhofer-Murray, 2025; Tuorto et al., 2018; Rashad, 2025; Dong et al., 2025). Positioned at the wobble position (position 34) of notable tRNA anticodons, Q modification considerably impacts codon-anticodon pairing efficiency and decoding accuracy (Ehrenhofer-Murray, 2025; Tuorto et al., 2018; Fergus et al., 2021). Unlike most RNA modifications, the biosynthesis of queuine is exclusive to bacteria and certain microorganisms, as eukaryotic cells are unable to produce this molecule on their own (Ehrenhofer-Murray, 2025; Tuorto et al., 2018; Rashad, 2025; Dong et al., 2025). Therefore, in mammals, queuine primarily depends on dietary sources and recovery from synthesis by gut bacteria (Ehrenhofer-Murray, 2025; Rashad, 2025). This feature makes Q-tRNA modification a plausible molecular interface between microbial metabolism and host translational regulation. Increasingly, evidence reveals that this “queuine-tRNA modification axis” between host and microbiota may comprise a novel metabolic-translational interface, allowing the microecological state to directly influence the host cell's protein translation program (Tuorto et al., 2018; Rashad, 2025; Morris and Elliott, 2001).

In a healthy state, queuine produced by the gut microbiota enters cells through the host's salvage system and is integrated into the anticodon loop of certain tRNAs, mediated by the eukaryotic tRNA-guanine transglycosylase complex composed of QTRT1/QTRT2, hence forming Q-tRNA (Fergus et al., 2021; Johannsson et al., 2018; Sievers et al., 2024). This process primarily focuses on tRNAs with the GUN anticodon, which includes tRNA^∧^Asp, tRNA^∧^Asn, tRNA^∧^His, and tRNA^∧^Tyr (Ehrenhofer-Murray, 2025; Fergus et al., 2021). The Q modification alters how efficiently these tRNAs decode codons like NAU/NAC, thereby affecting the speed and precision of the ribosome during translation elongation (Tuorto et al., 2018). Research shows that without Q modification, ribosomes are more likely to pause at significant codons, leading to higher translation error rates and issues with protein folding (Tuorto et al., 2018).

Q-tRNA works in conjunction with various other tRNA modifications to create synergistic networks. For example, the presence of queuine can enhance Dnmt2′s ability to methylate tRNA^∧^Asp at C38, which helps stabilize the tRNA structure and reduces translation mistakes (Tuorto et al., 2018). Furthermore, alterations like m^1^A, m5C, and τm5U contribute to the regulation of tRNA's structural stability and its ability to recognize codons (Kirchner and Ignatova, 2015; Motorin and Helm, 2022; Cappannini et al., 2024). Together, these modifications form a regulatory layer that supports translational fidelity and proteome stability, ensuring proteins fold properly by precisely controlling ribosomal pauses and speeds at different codons. Therefore, tRNA modifications are seen as an essential “translational quality control layer” that links genetic sequence data to protein functionality (Kirchner and Ignatova, 2015; Nedialkova and Leidel, 2015).

This translational buffering system may be particularly important for cells with high energetic demand. Neurons, especially dopaminergic neurons in the substantia nigra pars compacta (SNpc), are among the cell categories with the highest metabolic load in the human body (Surmeier et al., 2017; Ni and Ernst, 2022; Watanabe et al., 2024). These neurons have highly branched axonal structures, continuous autonomous firing activity, and extremely high mitochondrial energy demands, making their reliance on translational accuracy and proteostasis considerably greater than most other cell types (Surmeier et al., 2017; Ni and Ernst, 2022; Watanabe et al., 2024). Under healthy conditions, Q-tRNA and other tRNA modifications collectively maintain codon decoding stability and determine that mitochondrial, synaptic, and cytoskeletal proteins are synthesized with sufficient accuracy and efficiency to support the neuron's long-term high-energy metabolic state (Zheng et al., 2010; Chu and Kordower, 2015). If this traditional mitigation system stays stable, neurons can maintain a relatively stable proteome environment despite some oxidative stress or metabolic changes.

In addition to maintaining intracellular translational balance, the circulating RNA signaling network also stays relatively stable when conditions are healthy (Wang et al., 2018; Agris et al., 2017; Valadi et al., 2007; O'Brien et al., 2020; Turchinovich et al., 2016). Extracellular vesicles (EVs) and high-density lipoprotein (HDL) particles in the circulatory system carry different small RNAs, comprising miRNAs, rRNA fragments, and tRNA-derived RNAs (tRF/tDR; Valadi et al., 2007; O'Brien et al., 2020; Turchinovich et al., 2016). These RNA molecules are thought to take part in intercellular communication, playing roles in immune regulation, metabolic control, and tissue homeostasis maintenance. In steady-state conditions, circulating tRFs usually exhibit low and relatively stable expression levels, without generating significant inflammatory or stress signals (O'Brien et al., 2020). Therefore, in healthy organisms, a traditional regulatory framework is present, shaped by the balance of tRNA modifications and the stability of circulating RNA signals.

In general, under normal physiological conditions, the supply of queuine from the host and microbiota, along with Q-tRNA modification levels and other crucial tRNA modifications, form a complex system of translational homeostasis. This system offers cells an essential “translational buffering capacity” by improving codon decoding precision, maintaining steady translational kinetics, and reducing the production of misfolded proteins (Kirchner and Ignatova, 2015; Nedialkova and Leidel, 2015; Tuorto et al., 2018; Dong et al., 2025; Agris et al., 2007). At the same time, the small RNA signaling network in body fluids remains comparatively stable, offering a baseline context for inter-tissue information exchange (Wang et al., 2018; Agris et al., 2017; Valadi et al., 2007; O'Brien et al., 2020; Turchinovich et al., 2016). It is within this homeostatic framework that when queuine supply decreases, Q-tRNA modification declines, or the tRNA modification network becomes imbalanced, neuronal tolerance to metabolic pressure and oxidative stress may rapidly diminish, laying the groundwork for translational imbalance, mitochondrial impairment, and neurodegenerative pathology.

#### 3.6.2 Pathological State: “failure of translational buffering” in PD

In PD, several established pathological processes—including α-synuclein aggregation, mitochondrial dysfunction, oxidative stress, impaired proteostasis, and neuroinflammation—can place sustained pressure on translational homeostasis (Spillantini et al., 1997; Surmeier et al., 2017; Lashuel et al., 2013; Schapira, 2007; Wong and Krainc, 2017; Bose and Beal, 2016). Whether Q-tRNA modification is directly disrupted in this context remains unknown, but the PD environment provides a biologically plausible setting in which tRNA modification networks, tRF generation, and mitochondrial translational stress could become maladaptive. The typical pathological characteristics of PD include the aggregation of α-syn, mitochondrial dysfunction, ongoing neuroinflammation, and persistent oxidative stress (Spillantini et al., 1997; Lashuel et al., 2013; Schapira, 2007). Recent findings confirm that imbalances in gut microbiota and disruptions in the gut-brain axis are crucial in the development and advancement of PD (Sampson et al., 2016; Scheperjans et al., 2015; Bedarf et al., 2017). These factors are not independent, but form a mutually amplifying pathological network at the levels of cellular metabolism, proteostasis, and RNA regulation, progressively undermining the tRNA modification system that normally maintains translational stability (Surmeier et al., 2017; Lashuel et al., 2013; Schapira, 2007; Wong and Krainc, 2017; Bose and Beal, 2016; Sampson et al., 2016; Romano et al., 2021; Pakos-Zebrucka et al., 2016).

The aggregation of α-syn and mitochondrial damage significantly alter the cellular translation environment (Lashuel et al., 2013; Wong and Krainc, 2017; Mick et al., 2020; Pakos-Zebrucka et al., 2016). The presence of α-syn pathology can interfere with the transport of synaptic proteins and the dynamics of mitochondria, leading to protein folding stress and stress in the endoplasmic reticulum (Lashuel et al., 2013; Wong and Krainc, 2017). At the same time, impaired mitochondrial electron transport chain leads to reduced ATP, increased ROS, and metabolic imbalances, which can activate the ISR and other translational regulatory pathways, hence reprogramming cellular translation programs (Mick et al., 2020; Pakos-Zebrucka et al., 2016). In this persistently stressful setting, there is more ribosomal pausing, higher rates of translation errors, and a buildup of misfolded proteins, which further strain proteostasis (Kirchner and Ignatova, 2015; Nedialkova and Leidel, 2015; Mick et al., 2020; Pakos-Zebrucka et al., 2016; Tuorto et al., 2018). Since the tRNA modification network is a fundamental layer sustaining codon-decoding efficiency, when mitochondrial stress and translational burden persist, tRNA modification imbalance may progressively become a critical trigger for the collapse of the translational system (Kirchner and Ignatova, 2015; Nedialkova and Leidel, 2015; Mick et al., 2020; Pakos-Zebrucka et al., 2016; Tuorto et al., 2018; Agris et al., 2007).

Various elements might influence the queuine-Q-tRNA pathway in PD. Since queuine is primarily derived from gut microbiota and diet, changes in the microecology can affect its availability (Rashad, 2025). PD patients generally display modifications in gut microbiota composition, comprising a decrease in short-chain fatty acid-producing bacteria and an increase in inflammation-associated bacteria (Sampson et al., 2016; Romano et al., 2021). This change in the microecology affects both the immune and metabolic environments and might reduce the availability of queuine or its precursor molecules, leading to lower levels of Q-tRNA modification. Literature has previously reported that Q-tRNA deficiency leads to reduced codon-decoding efficiency, increased translational pausing, and protein folding abnormalities (Tuorto et al., 2018). Therefore, in the long-term pathological setting of PD, disruption of the microbiota-queuine-tRNA modification pathway might significantly contribute to the failure of the usual mitigating system to develop.

Simultaneously, the unusual buildup of tRFs in PD might worsen the imbalance in translation (Paldor et al., 2023; Madrer et al., 2025; Magee et al., 2019). Numerous lines of evidence have shown characteristic alterations in tRF profiles in the blood, cerebrospinal fluid, and substantia nigra tissue of PD patients, with several tRFs increased even before symptom onset (Madrer et al., 2025). These small RNAs are more than just degradation byproducts; they can also influence cellular translation through various mechanisms (Wang et al., 2018; Yamasaki et al., 2009; Emara et al., 2010; Sobala and Hutvagner, 2013). For instance, 5′-tiRNAs can inhibit translation onset and promote stress granule formation, hence modifying the cell's protein synthesis program (Yamasaki et al., 2009; Emara et al., 2010). Furthermore, specific tRFs can engage in inflammation and immune regulation through exosome-mediated intercellular communication, enhancing local inflammatory responses in neurodegenerative diseases (Dong et al., 2025). Therefore, in PD, tRF abnormalities might result from increased translational stress and also contribute to further translational suppression and proteostasis imbalance, creating a self-perpetuating pathological cycle.

An imbalance in the mitochondrial translation system further amplifies this mechanism (Bose and Beal, 2016; Peng et al., 2021; Yasukawa et al., 2001). Mitochondrial protein synthesis relies on the precise modification and stable structure of mt-tRNAs, and different abnormalities in mt-tRNA wobble site modifications have been reported to directly impact respiratory chain protein translation and cause mitochondrial diseases (Peng et al., 2021; Yasukawa et al., 2001). In Parkinson's disease models, there have been consistent observations of decreased mitochondrial translation efficiency and diminished activity of the respiratory chain complexes (Bose and Beal, 2016). If both cytoplasmic tRNA modifications and mitochondrial tRNA modifications are perturbed simultaneously, the cell not only experiences global translational burden, but also loses the capacity for protein synthesis necessary to maintain mitochondrial function (Tuorto et al., 2018; Dong et al., 2025; Wei et al., 2025; Peng et al., 2021; Yasukawa et al., 2001). This “dual translational burden” might be a significant factor causing PD neurons to consistently experience an energy crisis.

Ultimately, these molecular processes lead to a crucial occurrence at the cellular level: the selective susceptibility of nigral dopaminergic neurons. SNpc neurons have very high metabolic needs, continuous calcium-dependent firing, and intricate axonal networks, which makes them more reliant on mitochondrial function and proteostasis compared to other neuron types (Surmeier et al., 2017). When the translational buffering system is impaired, these neurons are impacted first (Surmeier et al., 2017; Kirchner and Ignatova, 2015; Nedialkova and Leidel, 2015; Ni and Ernst, 2022; Watanabe et al., 2024). Increased translation errors, insufficient mitochondrial protein synthesis, and persistent accumulation of stress granules may progressively undermine their ability to maintain synaptic function and energy metabolism (Surmeier et al., 2017; Kirchner and Ignatova, 2015; Nedialkova and Leidel, 2015; Peng et al., 2021; Yasukawa et al., 2001; Yamasaki et al., 2009; Emara et al., 2010; Sobala and Hutvagner, 2013). Eventually, the ongoing translational and metabolic imbalance results in a decline in neuronal function and cell death.

Therefore, the proposed model does not redefine PD as primarily a tRNA-modification disorder. Rather, it suggests that translational-homeostasis failure may represent an additional layer interacting with established PD mechanisms, including α-synuclein aggregation, mitochondrial impairment, oxidative stress, and neuroinflammation. This layer remains insufficiently validated and should be tested through direct profiling of Q-tRNA modification, tRF production, ribosomal pausing, and mitochondrial translation in PD-relevant tissues and models. In this process, α-syn aggregation, mitochondrial damage, inflammatory responses, and gut microbiota dysbiosis collectively impair Q-tRNA modification and the associated tRNA modification network, while abnormal tRF accumulation and mitochondrial translational imbalance further amplify translational burden. Ultimately, this sequence of events converts minor disruptions at the standard level into the targeted degeneration of nigral dopaminergic neurons, triggering the start and advancement of Parkinson's disease.

#### 3.6.3 Interventional state: the recalibrating effect of exercise on this axis

Despite the translational buffering system's increasing failure in PD, more evidence suggests that exercise can recalibrate this system through various mechanisms (Ahlskog, 2018; Petzinger et al., 2013; Pizzo et al., 2013; Wei et al., 2025; Lau et al., 2011; Gerecke et al., 2010). In clinical and animal studies, regular aerobic exercise not only enhances motor symptoms, but also slows dopaminergic neuron loss, reduces neuroinflammation, and enhances mitochondrial function (Ahlskog, 2018; Petzinger et al., 2013; Lau et al., 2011). These protective effects indicate that exercise influences not just the neuromuscular system, but also has the potential to alter the neurodegenerative pathological environment through metabolic, immune, and circulating signal networks. Integrating the “microbiota-queuine-tRNA modification axis” model posited earlier, it can be hypothesized that exercise may modulate this axis through at least three interrelated pathways: reshaping the peripheral metabolic environment, modifying circulating RNA signals, and influencing queuine availability via the gut microecology.

Exercise significantly alters the body's metabolic environment, thereby indirectly affecting RNA modifications and translation regulation (Li et al., 2024; Weber et al., 2025). During exercise, the rapid increase in skeletal muscle energy demand leads to marked elevations in lactate, TCA cycle intermediates, and different amino acid metabolites (Weber et al., 2025). These metabolic alterations suggest energy conversion processes and also function as signaling molecules involved in cellular regulation. For instance, current evidence has reported that aminoacyl-tRNA synthetases AARS1 and AARS2 can act as L-lactate sensors, directly binding lactate and participating in protein lactylation-related reactions (Li et al., 2024). This discovery shows that the translation system can directly detect metabolic changes caused by exercise. Although direct evidence that exercise considerably modifies Q-tRNA modification levels is recently lacking, the regulation of translation complexes and RNA modifying enzyme activities by metabolic signals offers a reasonable molecular basis for exercise to indirectly influence the tRNA modification landscape via the metabolic environment.

Physical activity can alter the makeup of small RNAs in the bloodstream, thereby affecting signals for communication between cells (Oliveira et al., 2018; Karvinen et al., 2023; Denham and Spencer, 2020). Circulating extracellular vesicles (EVs) and lipoprotein particles (such as HDL) are considered significant carriers for the propagation of small RNAs *in vivo*, comprising miRNAs, rRNA fragments, and tRNA-derived RNAs (tRF/tDR; O'Brien et al., 2020; Denham and Spencer, 2020). Studies have shown that both short-term and long-term exercise can alter the expression patterns of small RNAs in EVs and affect their distribution across different tissues (Denham and Spencer, 2020). For example, research on animals indicates that short-term aerobic exercise can alter the small RNA makeup in serum EVs, including tRNA-associated molecules (Oliveira et al., 2018); while in human studies, the relative abundance of tRNA-derived RNAs in HDL particles increased after exercise, revealing that exercise can redistribute RNA signals among various circulating carriers (Karvinen et al., 2023). tRFs have been shown to influence translation, the formation of stress granules, and inflammatory responses (Yamasaki et al., 2009; Emara et al., 2010; Sobala and Hutvagner, 2013), exercise-driven remodeling of circulating tRFs may comprise a novel global RNA signaling mechanism implicated in neuroprotection via peripheral-central communication.

Exercise might influence the availability of queuine by altering the gut's microbial environment (Ehrenhofer-Murray, 2025; Tuorto et al., 2018; Rashad, 2025; Dong et al., 2025; Wei et al., 2025; Allen et al., 2018; Mailing et al., 2019). Patients with Parkinson's disease often show changes in the composition of gut microbiota, which are closely associated with inflammation, metabolic issues, and neurological disorders (Sampson et al., 2016; Romano et al., 2021). At the same time, exercise is considered a profound lifestyle factor regulating the gut microecology (Allen et al., 2018; Mailing et al., 2019). Numerous studies determine that aerobic exercise can increase microbial diversity and boost the abundance of certain metabolism-related bacteria, hence modifying the production of short-chain fatty acids, bile acids, and other microbial metabolites (Allen et al., 2018; Mailing et al., 2019). In people with PD, exercise programs have also been shown to lead to changes in microbiota composition and reductions in inflammatory markers (Wei et al., 2025). As queuine is primarily produced by bacteria and salvaged by the host (Rashad, 2025), changes in the microbiota due to exercise might indirectly affect the availability of queuine or its precursors, thereby impacting Q-tRNA modification levels (Ehrenhofer-Murray, 2025; Tuorto et al., 2018; Rashad, 2025; Dong et al., 2025; Wei et al., 2025; Allen et al., 2018; Mailing et al., 2019).

If these pathways converge *in vivo*, exercise may have a translationally relevant recalibrating effect; however, this remains a hypothesis because no study has yet demonstrated that exercise-induced microbiota or metabolic remodeling directly changes Q-tRNA modification in PD. By improving systemic metabolism, reshaping circulating RNA carriers, and potentially altering microbiota-derived queuine/preQ1 availability, exercise could indirectly influence tRNA modification and translational stress. This possibility should be tested experimentally rather than assumed. In this situation, the kinetics of ribosomal translation, the environment for protein folding, and the efficiency of mitochondrial protein synthesis might all improve, thereby reducing the ongoing translational stress condition in PD. Animal studies also display that regular exercise can decrease α-syn pathological accumulation and increase mitochondrial function (Lau et al., 2011; Gerecke et al., 2010), further supporting the notion that exercise has global regulatory effects on neurodegenerative pathology.

In summary, according to the model proposed here, exercise can be viewed as an external intervention that can re-regulate the “microbiota-queuine-tRNA modification axis.” via three pathways—metabolic remodeling, modification of circulating RNA signals, and regulation of the gut microecology—exercise may partially reshape the Q-tRNA modification network and translational homeostasis, and decrease mitochondrial translational stress. This multi-layered regulatory process provides a novel molecular framework for determining the neuroprotective impacts of exercise in PD and offers significant clues for future investigation of intervention approaches targeting RNA modifications and small RNA signals.

#### 3.6.4 A state-dependent model: why exercise may be “protective,” “ineffective,” or even “maladaptive”

Although numerous studies determine that exercise has neuroprotective effects in PD, substantial interindividual variability has been observed in both clinical and animal experiments: several patients respond significantly to exercise interventions, while others display restricted enhancement or almost none (Ahlskog, 2011; Frazzitta et al., 2015). This diversity indicates that the neuroprotective effects of exercise might not adhere to a straightforward “dose-response” relationship, but are instead more dependent on the disease condition, metabolic background, and individual physiological variations. In the context of the proposed “microbiota-queuine-tRNA modification axis,” this variability can be explained through a model of translational regulation that depends on the state.

The molecular impact of exercise intervention may vary across different stages of PD. In the early or prodromal stage of the disease, although the neuronal translational buffering system is somewhat perturbed, it may retain some adaptive capacity; for instance, the tRNA modification network may not have been fully disrupted, and mitochondrial translation function may still have plasticity. At this stage, by enhancing the metabolic environment, decreasing inflammation, and enhancing mitochondrial function, exercise may help partially restore tRNA modification homeostasis and decrease translational burden, hence decreasing neuronal degeneration (Ahlskog, 2018; Petzinger et al., 2013). However, during the advanced phase of the disease, when α-syn aggregation, mitochondrial harm, and persistent inflammation are prevalent, it might be challenging to repair the structural damage to the translation system. Although exercise might still improve symptoms, its ability to alter translational homeostasis could be significantly reduced.

The molecular effects of exercise might also be influenced by its intensity and duration. Moderate exercise is believed to stimulate mitochondrial biogenesis, enhance metabolic efficiency, and boost neuroplasticity (Scarpulla, 2011). However, too much exercise can lead to increased oxidative stress and inflammation, putting extra pressure on neurons (Radak et al., 2008). From a translational regulation aspect, moderate exercise may decrease translational stress by enhancing the metabolic environment, whereas overly intense exercise could further activate stress response pathways (such as the ISR), possibly exacerbating translational suppression or tRF production. Hence, exercise intensity may shape its net effect on the tRNA modification network and translation system.

The background of gut microbiota might also play a crucial role in affecting the outcomes of exercise (Klein and Westenberger, 2012). PD patients generally display gut microbiota dysbiosis, and microbiota structure varies considerably among individuals (Romano et al., 2021). Because queuine availability relies on gut microbiota and diet, alterations in microbiota composition might directly affect Q-tRNA modification levels (Rashad, 2025). If exercise can facilitate the recovery of beneficial bacteria and increase queuine supply, it may strengthen translational homeostasis; In contrast, if the microbiota environment cannot support queuine synthesis or utilization, the regulatory effect of exercise on this axis may be constrained. Recent research indicates that the improvement of PD symptoms through exercise might be linked to metabolic signals from the microbiota (Wei et al., 2025; Allen et al., 2018).

Genetic background may also influence the influence of exercise interventions (Klein and Westenberger, 2012). Different genetic subtypes of PD (such as LRRK2, PINK1, Parkin, or GBA mutations) involve mitochondrial function, protein degradation, or cellular stress responses (Klein and Westenberger, 2012). These genetic elements might alter how neurons respond to translational stress, thereby affecting how exercise can adjust the translation system. For instance, in a genetic background with impaired mitochondrial function, exercise may more readily exhibit protective effects by enhancing mitochondrial metabolism, whereas in a background with impaired protein degradation systems, translational regulation may become a more critical intervention node.

Based on these factors, an “adaptive window” model can be proposed: the protective benefits of exercise on the translation system have limits and are most effective within a specific range. When the disease is in an early stage, exercise intensity is moderate, the microbiota can offer sufficient queuine, and the neuronal translation system still has several plasticity, exercise can, via metabolic and RNA signal remodeling, pull the system back toward a comparatively stable translational state. During this period, the tRNA modification network, mitochondrial translation, and protein homeostasis can be somewhat reestablished, thereby reducing the stress on nigral dopaminergic neurons. Nevertheless, when the disease progresses to a more severe stage or the exercise stimulus is significant, this regulatory process may fail or even backfire, causing further exacerbation of translational stress.

This adaptive-window model also provides a framework for defining measurable predictors of exercise responsiveness. Candidate clinical and physiological parameters include baseline disease stage, MDS-UPDRS III score, non-motor symptom burden, levodopa equivalent daily dose, autonomic dysfunction, constipation severity, cardiorespiratory fitness, and habitual physical activity. Recent clinical evidence suggests that individual PD phenotypes may influence clinical improvement after exercise, supporting the need to move beyond uniform exercise prescriptions toward stratified intervention models (Lee et al., 2025). Autonomic dysfunction and baseline physical activity may also shape cardiorespiratory responses to exercise in PD, indicating that physiological reserve should be considered when predicting exercise responsiveness (Veldkamp et al., 2025). In parallel, microbiota-related parameters, including gut microbiota diversity, constipation severity, and fecal or circulating queuine/preQ1 levels, may be relevant because aerobic exercise has been shown to improve clinical symptoms and modulate gut microbiota/metabolic profiles in people with PD (Wei et al., 2025).

At the molecular level, inflammatory markers, mitochondrial stress markers, blood/CSF tRF signatures, EV/HDL-associated small RNA cargo, and Q-tRNA modification status may provide additional candidate predictors. PD-related tRF signatures have been reported in blood and CSF, and recent work suggests that repetitive-sequence tRF profiles may help identify pre-symptomatic PD after further validation (Paldor et al., 2023; Madrer et al., 2025). Exercise-responsive circulating RNA carriers may also be relevant, because acute exercise can differentially alter EV- and HDL-associated small RNAs, including increased transfer RNA-derived small RNAs in HDL particles (Karvinen et al., 2023). However, Q-tRNA modification status and queuine/preQ1 balance should be treated as exploratory biomarkers rather than established predictors of exercise response, because no study has yet shown that exercise modifies Q-tRNA levels in PD. Future exercise trials should therefore stratify participants before intervention and test whether clinical phenotype, physiological reserve, microbiota composition, tRF profiles, queuine/preQ1 levels, and Q-tRNA modification status predict motor, non-motor, and molecular outcomes.

### 3.7 Clinical implications: from molecular framework to precision diagnosis and intervention

Building upon this conceptual framework, the following sections explore its tangible clinical applications. By integrating tRF profiles, Q-tRNA modification status, microbiota-derived metabolites, and exercise-response phenotypes, this framework may eventually support more precise PD stratification. However, these applications remain investigational. At present, tRF signatures have stronger biomarker support than Q-tRNA modification status in PD, whereas Q-tRNA-based intervention remains a mechanistic hypothesis requiring validation (Paldor et al., 2023; Madrer et al., 2025; Magee et al., 2019). The clinical translation of this framework begins with its potential to refine how patients are identified and classified (Table 2).

**Table 2:** Potential clinical and experimental implications of the tRF/Q-tRNA modification framework in PD.

Core concepts and mechanisms	Key molecular indicators/targets	Clinical/research significance	Supporting excerpts
Potential molecular stratification based on tRF profiles, Q-tRNA modification status, queuine/preQ1 levels, and microbiota features.	tRFs (e.g., RGTTCRA-tRFs ↑, MT-tRFs ↓) Q-tRNA modification levels Queuine levels	- Differentiate PD patients from healthy controls.	Paldor et al., 2023; Madrer et al., 2025; Magee et al., 2019
		- Identify high-risk individuals in the pre-symptomatic stage.	
		- Reveal disease heterogeneity (e.g., “translational stress-dominant subtype” vs. “inflammation/mitochondrial defect subtype”).	
		- Shift from traditional symptom-based classification to molecular subtyping.	
Hypothesis-driven prediction of exercise responsiveness based on clinical phenotype, exercise capacity, microbiota composition, tRF signatures, and candidate Q-tRNA modification status.	Translational system plasticity (high Q-tRNA modification, low tRF aberrations) Degree of translational system collapse (severe tRF accumulation, mitochondrial translational imbalance)	Responders: Exercise may be more likely to improve clinical outcomes and engage metabolic/microbiota-linked protective pathways. Non-responders: May require integration with anti-inflammatory, mitochondrial protection, or microbiota-targeted therapies, rather than a uniform exercise prescription.	Petzinger et al., 2013; Ahlskog, 2011
Experimental targeting of the queuine/QTRT/Q-tRNA pathway to test whether translational homeostasis and mitochondrial stress can be modified.	Queuine supplementation (derived from diet/microbiota) QTRT1/QTRT2 enzyme activity tRNA modification enzyme modulators	Potential experimental readouts: α-synuclein burden, oxidative stress, mitochondrial respiration, ribosomal pausing, tRF generation, Q-tRNA abundance, and dopaminergic neuronal survival. Therapeutic pathways: (1) Dietary/pharmaceutical supplementation of queuine precursors. (2) Modulation of QTRT enzyme activity. (3) Development of small molecule drugs targeting tRNA modification enzymes.	Kirchner and Ignatova, 2015; Tuorto et al., 2018; Rashad, 2025; Lin et al., 2024; Sievers et al., 2021
Testing whether combined exercise, microbiota-targeted intervention, and RNA-biology approaches can jointly influence neuronal homeostasis.	Exercise (improves metabolic environment) Microecological intervention (reshapes queuine supply) RNA targeting (regulates tRF production/translational homeostasis)	Synergistic effects: (1) Exercise improves systemic metabolism. (2) Microecological intervention (probiotics/diet) reshapes queuine supply. (3) RNA targeting restores the translation system. Personalized treatment: Match optimal intervention combinations based on molecular subtyping (tRF profiles, Q-tRNA status, and microbiota composition).	Ahlskog, 2018; Petzinger et al., 2013; Allen et al., 2018; Mailing et al., 2019; Wiener and Schwartz, 2021

#### 3.7.1 Potential for stratified diagnosis based on tRF/Q-tRNA profiles

Small RNAs, especially tRFs, have increasingly been investigated as candidate biomarkers for neurodegenerative diseases (Paldor et al., 2023; Madrer et al., 2025; Magee et al., 2019; Wang et al., 2018). In PD research, several independent cohorts have reported characteristic tRF expression changes in blood, cerebrospinal fluid (CSF), and brain tissue, with several tRFs increased even before symptom onset (Paldor et al., 2023; Magee et al., 2019; Madrer et al., 2025). For example, recent evidence reported that a group of RGTTCRA-tRFs carrying a conserved sequence motif were considerably increased in the substantia nigra, CSF, and peripheral blood of PD patients, while mitochondria-derived tRFs (MT-tRFs) reported a decreasing trend (Madrer et al., 2025). An index based on these two tRF classes could differentiate PD patients from healthy individuals and detect high-risk people before symptoms appear, showing promise for early diagnosis (Madrer et al., 2025).

Compared to traditional protein biomarkers, tRFs provide several benefits (Wang et al., 2018; Agris et al., 2017; O'Brien et al., 2020). They are relatively stable in bodily fluids and can be carried over long distances via extracellular vesicles (EVs) or lipoprotein particles (O'Brien et al., 2020). Moreover, because tRFs are linked to the translation system, they may reflect cellular stress states and modifications in translational homeostasis (Wang et al., 2018; Yamasaki et al., 2009; Emara et al., 2010; Sobala and Hutvagner, 2013). Furthermore, changes in tRF expression are often closely associated with cellular metabolism, inflammatory responses, and mitochondrial function, which are all crucial elements of PD pathology (Yamasaki et al., 2009; Emara et al., 2010; Sobala and Hutvagner, 2013). Hence, tRFs can function not only as disease markers, but also as molecular indicators of possible translational stress.

In the “microbiota-queuine-tRNA modification axis” model posited herein, tRFs and Q-tRNA modification can collectively comprise a novel framework for stratified diagnosis. For instance, by integrating the detection of blood tRF profiles, queuine levels, and Q-tRNA modification status, it may be possible to determine various molecular subtypes of PD. Furthermore, integrating molecular subtyping with a patient's exercise response, gut microbiota profile, and genetic makeup could lead to a more refined disease classification system. For instance, several patients may display profound tRF elevation and reduced Q-tRNA modification, possibly denoting a translational stress-dominant PD subtype; others may be defined mainly by inflammation or mitochondrial defects. Thus, the tRF/Q-tRNA axis might be useful not only for early diagnosis but also as an essential tool for identifying PD heterogeneity. As small RNA sequencing technologies and epitranscriptomics advance, future research is expected to evaluate tRNA modification profiles and tRF expression maps in PD patients with greater precision. These technologies will help create disease classification models based on the status of translation systems, enabling a shift in PD diagnosis from traditional symptom-based classification to molecular subtyping.

#### 3.7.2 A precision intervention framework according to exercise responsiveness

In clinical practice, exercise has been extensively suggested as a profound adjunctive therapy for PD (Ahlskog, 2018; Petzinger et al., 2013; Pizzo et al., 2013). Nevertheless, there is profound individual variability in patients' responses to exercise interventions; several patients obtain significant neuroprotective effects, while others display restricted enhancement (Petzinger et al., 2013; Ahlskog, 2011). This variability indicates that exercise programs might require more tailored approaches rather than a one-size-fits-all prescription (Young and Wek, 2016; Ivanov et al., 2011; Ahlskog, 2011; Frazzitta et al., 2015). Within the molecular framework posited herein, tRF/Q-tRNA modification status could function as significant molecular indicators for forecasting exercise responsiveness. For instance, if the translation system of certain patients still has several plasticity, such as comparatively high Q-tRNA modification levels and minor tRF modifications, then exercise may more readily reshape translational homeostasis by enhancing the metabolic environment and microbiota structure, hence exhibiting neuroprotective effects. On the other hand, if patients have already shown a significant breakdown of the translation system, like severe abnormal tRF buildup and mitochondrial translation imbalance, the impact of exercise intervention might be relatively restricted.

Hence, in future studies, an exercise intervention framework based on molecular subtyping could be attempted, where patients are categorized into various subtypes based on their tRF expression profile, queuine levels, and Q-tRNA modification status, and their response to exercise intervention is predicted. Therefore, in this context, patients could be categorized as “exercise responders” and “non-responders.” The former may have better translation system plasticity and respond significantly to exercise intervention; the latter may need integration with other therapeutic modalities, such as anti-inflammatory treatment, mitochondrial protection approaches, or microbiota-targeted interventions.

Furthermore, incorporating gut microbiota analysis might enhance predictive accuracy. For instance, if a patient's microbiota structure can encourage queuine synthesis and metabolism, exercise may more readily exhibit effects through the microbiota-queuine-tRNA modification axis. In contrast, if the microbiota environment is markedly dysbiotic, probiotics, dietary modifications, or microecological therapies may be required to reshape the function of this axis. Via such a multi-dimensional subtyping approach, translational experimental exercise prescriptions could be converted into mechanism-based precision exercise therapy.

As tRNA modificationomics, small RNA biology, and microecology research advance, future PD interventions might evolve from single treatments to complex, multi-faceted strategies. Within this framework, exercise is not only a lifestyle intervention, but could also turn into a promising tool for regulating the translation system, reshaping tRNA modification homeostasis, and decreasing mitochondrial stress. Incorporating the tRF/Q-tRNA axis into the disease diagnosis and treatment framework is likely to usher in a new era of precision medicine evidence in PD.

#### 3.7.3 Potential for queuine supplementation or Q-tRNA targeted intervention

As comprehension of the influence of tRNA modifications in neurodegenerative diseases deepens, the Q-tRNA modification axis is increasingly considered a possible therapeutic target (Kirchner and Ignatova, 2015; Tuorto et al., 2018; Rashad, 2025; Dong et al., 2025; Agris et al., 2007). Since queuine mainly originates from the gut microbiota and diet and is integrated into the anticodon site of certain tRNAs through QTRT1/QTRT2 catalysis, its level is possibly controllable, making this axis theoretically amenable to intervention (Rashad, 2025). Recent research indicates that queuine is involved in regulating translational balance and may also have a direct impact on neuron survival and mitochondrial activity (Tuorto et al., 2018; Dong et al., 2025; Lin et al., 2024; Wei et al., 2025).

In models of neurodegenerative diseases, studies have reported several neuroprotective effects of queuine. For instance, in cell and animal model studies, queuine or its derivatives could decrease mitochondrial impairment, decrease oxidative stress levels, and increase neuronal survival (Lin et al., 2024; Sievers et al., 2021). Furthermore, various studies have indicated that queuine treatment can reduce α-syn aggregation and its related toxicity, suggesting that this molecule might influence PD pathology by affecting translational homeostasis or mitochondrial function (Sievers et al., 2021). Even though this evidence is still in the preclinical phase, the findings provide significant insights into the potential use of queuine for neurodegenerative diseases.

From a molecular perspective, queuine might function through various pathways. Supplementing with queuine can alter Q-tRNA modification levels, thereby enhancing codon-decoding efficiency and reducing translation errors (Tuorto et al., 2018). In addition, Q-tRNA modification can impact protein folding and protein quality control, hence decreasing the accumulation of misfolded proteins and stress responses (Kirchner and Ignatova, 2015). Furthermore, recent findings have shown a significant connection between queuine and mitochondrial function; for example, in models associated with mt-tRNA mutations, queuine can reduce mitochondrial translation defects and alter energy metabolism (Lin et al., 2024). These outcomes collectively recognize that the queuine-Q-tRNA axis may exhibit global effects on neuronal homeostasis by integrating the translation system and mitochondrial function.

Future therapeutic strategies might focus on modulating the Q-tRNA axis through various methods. For example, one approach is to enhance the availability of queuine or its precursors through dietary supplementation or pharmaceutical forms, hence increasing Q-tRNA modification levels. An alternative method is to directly influence QTRT1/QTRT2 activity to aid in the formation of Q modifications (Fergus et al., 2021; Johannsson et al., 2018; Sievers et al., 2024). In addition, with the development of RNA modificationomics and small molecule drug screening technologies, targeted modulators for tRNA modifying enzymes may also be progressed in future studies (Cappannini et al., 2024; Wiener and Schwartz, 2021).

To sum up, the queuine/QTRT axis offers a set of RNA modification-based therapeutic strategies that differ from traditional targets like neurotransmitters or protein aggregation. Evidence in this area is still in its early stages, but holds possible value of investigation.

#### 3.7.4 Integration approaches: exercise + microecological intervention + RNA biology targeting

Despite advancements in individual treatments for PD, the disease encompasses various pathological processes, including protein aggregation, mitochondrial dysfunction, inflammation, and imbalances in translational homeostasis (Spillantini et al., 1997; Surmeier et al., 2017; Lashuel et al., 2013; Schapira, 2007; Wong and Krainc, 2017; Bose and Beal, 2016). Hence, future treatment paradigms will similarly need multi-layered integration interventions. Within the framework of the “microbiota-queuine-tRNA modification axis” posited herein, an encouraging approach is to integrate exercise, microecological modulation, and RNA biology targeting to reshape neuronal homeostasis at numerous levels.

As a worldwide intervention, exercise can enhance the metabolic environment, boost mitochondrial function, and reduce inflammatory responses (Ahlskog, 2018; Petzinger et al., 2013). According to the model in our review, exercise might impact the tRNA modification network and translational balance by altering circulating small RNA signals and the gut's microecological structure. Additionally, interventions targeting the gut microbiota, such as dietary modifications, probiotics, or microecological therapies, could further increase the supply potential of queuine and reshape host-microbe metabolic interactions (Allen et al., 2018; Mailing et al., 2019). Because queuine relies on microbial metabolism, interventions targeting the microbiota could directly affect Q-tRNA modification levels (Ehrenhofer-Murray, 2025; Tuorto et al., 2018; Rashad, 2025; Dong et al., 2025). Based on this foundation, RNA biology targeting approaches could further increase the regulatory capacity of this axis. For instance, regulating tRNA modifying enzyme activity, inhibiting pathological tRF production, or reshaping translational homeostasis could help decrease neuronal translational stress (Kirchner and Ignatova, 2015; Dong et al., 2025; Yamasaki et al., 2009; Emara et al., 2010; Sobala and Hutvagner, 2013; Wiener and Schwartz, 2021). Recently, drug development targeting RNA modifications (epitranscriptomic targets) has emerged as a new evidence direction; for example, modulators targeting mRNA methylases or RNA-binding proteins are progressively entering preclinical evidence stages (Wiener and Schwartz, 2021). In future studies, similar methods might be applied to the tRNA modification system.

Therefore, a possible future therapeutic approach could be: Exercise enhances the overall metabolic environment, followed by microecological intervention adjusting queuine supply, and targeting RNA biology to restore the translation system. This multi-layered integration approach aims to concurrently target numerous pathological nodes in PD, possibly generating synergistic effects. In addition to major interventions aimed at translational homeostasis, supplementary strategies addressing oxidative stress and mitochondrial dysfunction could also be considered. For example, inhaling molecular hydrogen has been investigated as a potential supportive treatment for Parkinson's Disease, though the recent evidence is largely theoretical and based on individual cases rather than definitive molecular proof (Ichikawa et al., 2024). Moreover, this approach also holds potential for individualized treatment. For example, by evaluating a patient's tRF expression profile, Q-tRNA modification status, and gut microbiota composition, it is possible to identify patient subgroups best suited for different intervention strategies. Several patients may be more suitable for exercise intervention, while others may count more on microecological or RNA-targeted therapies. This comprehensive treatment approach, grounded in molecular subtyping, shows potential for advancing PD therapy from traditional symptom management to precision medicine based on underlying mechanisms.

Overall, integrating exercise, microecological, and RNA biology targeting approaches offer a novel conceptual framework for PD treatment. This joint intervention addresses the increasing pathological aspect of neuronal translational balance and could provide new perspectives for the advancement of multi-target therapies.

### 3.8 Knowledge gaps and experimental priorities

Although the exercise-microbiota-queuine-tRNA modification axis is conceptually attractive, several links remain untested. Addressing these gaps is essential before this framework can be considered mechanistic or clinically actionable.

#### 3.8.1 Direct profiling of Q-tRNA modification in PD

The first priority is to determine whether Q-tRNA modification is altered in PD. Future studies should measure Q-tRNA levels in postmortem SNpc, striatum, peripheral blood mononuclear cells, plasma EVs, CSF, and patient-derived iPSC dopaminergic neurons. Suitable methods include LC-MS/MS-based tRNA modification profiling, APB northern blotting for Q-modified tRNAs, tRNA modification sequencing, and QTRT1/QTRT2 expression/activity assays (Cappannini et al., 2024). These measurements should be correlated with α-synuclein burden, mitochondrial respiratory function, oxidative stress, ISR markers, and tRF profiles. Without such data, Q-tRNA deficiency should remain a candidate mechanism rather than a confirmed PD feature.

#### 3.8.2 Testing whether exercise modifies Q-tRNA levels in PD models

The second priority is to test whether exercise alters Q-tRNA modification *in vivo*. A feasible design would use MPTP, 6-OHDA, α-synuclein PFF, or genetic PD models randomly assigned to sedentary control, moderate-intensity exercise, high-intensity exercise, and exercise plus microbiota manipulation (Citron et al., 2025; Lau et al., 2011; Gerecke et al., 2010; Klein and Westenberger, 2012). Q-tRNA modification should be measured in SNpc, striatum, blood, and fecal metabolite-linked compartments before and after intervention. Key outcomes should include Q-tRNA abundance, QTRT1/QTRT2 expression, queuine/preQ1 levels, tRF signatures, mitochondrial respiration, TH-positive neuronal survival, striatal dopamine content, neuroinflammatory markers, and motor behavior. This design would directly address whether exercise-induced neuroprotection is accompanied by Q-tRNA remodeling.

#### 3.8.3 Microbiota-queuine causality

The third priority is to establish whether gut microbiota causally regulates host Q-tRNA modification in PD-relevant contexts. Antibiotic depletion, germ-free mice, fecal microbiota transplantation, defined bacterial consortia, queuine supplementation, and preQ1 manipulation could be combined with PD models (Sampson et al., 2016; Shan et al., 2025; Zhang et al., 2025). A stepwise design should test whether microbiota manipulation changes fecal/plasma queuine and preQ1, whether these changes alter Q-tRNA modification in peripheral and neural tissues, and whether Q-tRNA changes influence mitochondrial stress and dopaminergic neuronal vulnerability. This would clarify whether microbiota-derived queuine is merely associated with or causally involved in translational stress adaptation.

#### 3.8.4 Functional consequences of preQ1-queuine competition

Recent evidence indicates that preQ1 can compete with queuine for host tRNA modification sites and influence mammalian translation quality control (Zhang et al., 2025). Future PD studies should quantify queuine/preQ1 ratios in feces, plasma, CSF, and PD model tissues. They should also test whether altered preQ1/queuine balance changes codon-specific translation, ribosomal pausing, unfolded protein response activation, mitochondrial function, and neuronal stress tolerance. These experiments would determine whether PD-associated dysbiosis affects not only queuine supply but also competitive microbial metabolite pressure on host tRNA modification.

#### 3.8.5 ANG/tRNA cleavage switch

The ANG/tRNA cleavage pathway requires mechanistic dissection in PD (Yamasaki et al., 2009; Emara et al., 2010; Sobala and Hutvagner, 2013; Prehn and Jirström, 2020). Future studies should determine whether acute vs. chronic mitochondrial stress produces different ANG localization patterns, tiRNA/tRF repertoires, stress-granule dynamics, and translational outcomes. Experiments should manipulate ANG activity, ribonuclease inhibitor levels, autophagy, and oxidative stress in dopaminergic neurons and microglia. Time-course experiments are especially important because transient tiRNA production may be adaptive, whereas chronic tRF accumulation may promote sustained translational repression and inflammatory signaling (Yamasaki et al., 2009; Emara et al., 2010; Sobala and Hutvagner, 2013; Prehn and Jirström, 2020).

#### 3.8.6 EV/HDL-mediated peripheral-to-central RNA communication

The transport of exercise-responsive EV- and HDL-associated tRFs into the CNS remains unresolved (Karvinen et al., 2023; Suzuki et al., 2020; Agris et al., 2017). Future studies should use labeled EVs, HDL-tracking approaches, BBB transwell models, endothelial-cell uptake assays, and *in vivo* brain-region mapping to determine whether peripheral tRFs enter the brain, remain at the neurovascular interface, or act indirectly through immune and vascular pathways. Recipient-cell specificity should be tested in neurons, astrocytes, microglia, endothelial cells, and pericytes. These experiments are necessary before circulating tRFs can be interpreted as direct peripheral-to-brain effector molecules.

#### 3.8.7 Clinical stratification of exercise responders and non-responders

Future clinical trials should test whether tRF/Q-tRNA/microbiota profiles predict exercise responsiveness. Participants should be stratified by disease stage, MDS-UPDRS III score, non-motor symptoms, levodopa equivalent daily dose, constipation severity, autonomic dysfunction, baseline cardiorespiratory fitness, habitual physical activity, inflammatory markers, microbiota diversity, queuine/preQ1 levels, tRF signatures, and Q-tRNA modification status (Young and Wek, 2016; Ivanov et al., 2011). Longitudinal sampling before, during, and after exercise intervention would allow investigators to distinguish baseline predictors from treatment-induced molecular changes. This design would help determine whether the proposed axis has practical value for precision exercise prescription.

## 4 Conclusion and Perspectives

This review proposes the exercise-microbiota-queuine-tRNA modification axis as a hypothesis-generating framework for understanding how systemic metabolic remodeling, microbial metabolism, RNA modification, and translational stress may intersect in PD. The strongest evidence supports individual components of the model, including microbial/dietary queuine supply, QTRT1/QTRT2-mediated Q-tRNA modification, Q-tRNA effects on codon decoding and proteostasis, PD-associated tRF signatures, exercise-induced microbiota remodeling, and exercise-responsive changes in circulating RNA carriers. However, the complete causal sequence linking exercise to microbiota-derived queuine/preQ1 balance, Q-tRNA remodeling, mitochondrial translational recalibration, and protection of SNpc dopaminergic neurons remains unproven. Therefore, this framework should not be interpreted as an established disease mechanism, but as a structured roadmap for future investigation. Direct Q-tRNA profiling in PD, exercise-intervention studies with tRNA modification endpoints, microbiota/queuine manipulation, and mechanistic testing of EV/HDL RNA transport are now required. If validated, this axis may provide new opportunities for molecular stratification, biomarker development, and precision exercise interventions in PD.

## References

[B1] Agris P. F. Narendran A. Sarachan K. Väre V. Y. P. Eruysal E. (2017). The role of RNA modifications in translational fidelity. Enzymes. 41, 1–50. doi: 10.1016/bs.enz.2017.03.005 28601219 PMC8118379

[B2] Agris P. F. Vendeix F. A. Graham W. D. (2007). tRNA's wobble decoding of the genome: 40 years of modification. J. Mol. Biol. 366, 1–13. doi: 10.1016/j.jmb.2006.11.046 17187822

[B3] Ahlskog J. E. (2011). Does vigorous exercise have a neuroprotective effect in Parkinson disease? Neurology 77, 288–294. doi: 10.1212/WNL.0b013e318225ab66 21768599 PMC3136051

[B4] Ahlskog J. E. (2018). Aerobic exercise: evidence for a direct brain effect to slow Parkinson disease progression. Mayo Clin. Proc. 93, 360–372. doi: 10.1016/j.mayocp.2017.12.015 29502566

[B5] Allen J. M. Mailing L. J. Niemiro G. M. Moore R. Cook M. D. White B. A. . (2018)., Exercise alters gut microbiota composition and function in lean and obese humans. Med. Sci. Sports Exerc. 50, 747–757. doi: 10.1249/MSS.0000000000001495 29166320

[B6] Baethge C. Goldbeck-Wood S. Mertens S. (2019). SANRA—a scale for the quality assessment of narrative review articles. Res. Integr. Peer Rev. 4:5. doi: 10.1186/s41073-019-0064-8 30962953 PMC6434870

[B7] Baindoor S. Gibriel H. A. Y. Venø M. T. Su J. Morrissey E. P. Jirström E. . (2024). Distinct fingerprints of tRNA-derived small non-coding RNA in animal models of neurodegeneration. Dis. Model Mech. 17:dmm050870. doi: 10.1242/dmm.050870 39552337 PMC11603119

[B8] Banks W. A. Sharma P. Bullock K. M. Hansen K. M. Ludwig N. Whiteside T. L. (2020). Transport of extracellular vesicles across the blood-brain barrier: brain pharmacokinetics and effects of inflammation. Int. J. Mol. Sci. 21:4407. doi: 10.3390/ijms21124407 32575812 PMC7352415

[B9] Bedarf J. R. Hildebrand F. Coelho L. P. Sunagawa S. Bahram M. Goeser F. . (2017). Functional implications of microbial and viral gut metagenome changes in early stage L-DOPA-naïve Parkinson's disease patients. Genome Med. 9:39. doi: 10.1186/s13073-017-0428-y 28449715 PMC5408370

[B10] Begley U. Dyavaiah M. Patil A. Rooney J. P. DiRenzo D. Young C. M. . (2007). Trm9-catalyzed tRNA modifications link translation to the DNA damage response. Mol. Cell. 28, 860–870. doi: 10.1016/j.molcel.2007.09.021 18082610 PMC2211415

[B11] Bose A. Beal M. F. (2016). Mitochondrial dysfunction in Parkinson's disease. J. Neurochem. 139, 216–231. doi: 10.1111/jnc.13731 27546335

[B12] Cappannini A. Ray A. Purta E. Mukherjee S. Boccaletto P. Moafinejad S. N. . (2024). MODOMICS: a database of RNA modifications and related information. 2023 update. Nucleic Acids Res. 52, D239–D244. doi: 10.1093/nar/gkad1083 38015436 PMC10767930

[B13] Chakrabarty Y. Yang Z. Chen H. Chan D. C. (2024). The HRI branch of the integrated stress response selectively triggers mitophagy. Mol. Cell. 84, 1090.e6–1100.e6. doi: 10.1016/j.molcel.2024.01.016 38340717 PMC11062084

[B14] Cheng J. Williams J. P. Zhou L. Wang P. C. Sun L. N. Li R. H. . (2024). Ozone rectal insufflation mitigates chronic rapid eye movement sleep deprivation-induced cognitive impairment through inflammation alleviation and gut microbiota regulation in mice. Med. Gas Res. 14, 213–224. doi: 10.4103/mgr.MEDGASRES-D-23-00036 39073330 PMC11257187

[B15] Chu Y. Kordower J. H. (2015). The prion hypothesis of Parkinson's disease. Curr. Neurol. Neurosci. Rep. 15:28. doi: 10.1007/s11910-015-0549-x 25868519

[B16] Citron B. A. Guzman M. Shapdoor B. Rameshwar P. Sokratian A. Shaikh A. L. . (2025). Moderate chronic treadmill exercise slows dopaminergic neuron loss in a rat model of Parkinson's disease and alters RNA content of circulating plasma exosomes. J. Neurosci. Res. 103:e70084. doi: 10.1002/jnr.70084 41078024 PMC12516300

[B17] Denham J. Spencer S. J. (2020). Emerging roles of extracellular vesicles in the intercellular communication for exercise-induced adaptations. Am. J. Physiol. Endocrinol. Metab. 319, E320–E329. doi: 10.1152/ajpendo.00215.2020 32603601

[B18] Dong X. Li Q. Li R. Li Y. Jin F. Li H. . (2025). Inhibition of tRF-02514 in extracellular vesicles preserves microglia pyroptosis and protects against Parkinson's disease. Mol. Neurobiol. 62, 11047–11063. doi: 10.1007/s12035-025-04925-2 40254704 PMC12367836

[B19] Ehrenhofer-Murray A. E. (2025). Queuine: a bacterial nucleobase shaping translation in eukaryotes. J. Mol. Biol. 437:168985. doi: 10.1016/j.jmb.2025.168985 39956693

[B20] Emara M. M. Ivanov P. Hickman T. Dawra N. Tisdale S. Kedersha N. . (2010). Angiogenin-induced tRNA-derived stress-induced RNAs promote stress-induced stress granule assembly. J. Biol. Chem. 285, 10959–10968. doi: 10.1074/jbc.M109.077560 20129916 PMC2856301

[B21] Fergus C. Al-Qasem M. Cotter M. McDonnell C. M. Sorrentino E. Chevot F. . (2021). The human tRNA-guanine transglycosylase displays promiscuous nucleobase preference but strict tRNA specificity. Nucleic Acids Res. 49, 4877–4890. doi: 10.1093/nar/gkab289 34009357 PMC8136771

[B22] Frazzitta G. Maestri R. Bertotti G. Riboldazzi G. Boveri N. Perini M. . (2015). Intensive rehabilitation treatment in early Parkinson's disease: a randomized pilot study with a 2-year follow-up. Neurorehabil. Neural Repair 29, 123–131. doi: 10.1177/1545968314542981 25038064

[B23] Gerecke K. M. Jiao Y. Pani A. Pagala V. Smeyne R. J. (2010). Exercise protects against MPTP-induced neurotoxicity in mice. Brain Res. 1341, 72–83. doi: 10.1016/j.brainres.2010.01.053 20116369 PMC2884060

[B24] Ichikawa Y. Sato B. Hirano S. Takefuji Y. Satoh F. (2024). Realizing brain therapy with “smart medicine”: mechanism and case report of molecular hydrogen inhalation for Parkinson's disease. Med. Gas Res. 14:8. doi: 10.4103/2045-9912.385949 39073335 PMC466992

[B25] Ivanov P. Emara M. M. Villen J. Gygi S. P. Anderson P. (2011). Angiogenin-induced tRNA fragments inhibit translation initiation. Mol. Cell. 43, 613–623. doi: 10.1016/j.molcel.2011.06.022 21855800 PMC3160621

[B26] Johannsson S. Neumann P. Ficner R. (2018). Crystal structure of the human tRNA guanine transglycosylase catalytic subunit QTRT1. Biomolecules 8:81. doi: 10.3390/biom8030081 30149595 PMC6165067

[B27] Karvinen S. Korhonen T. Sievänen T. Karppinen J. E. Juppi H. Jakoaho V. . (2023). Extracellular vesicles and high-density lipoproteins: exercise and oestrogen-responsive small RNA carriers. J. Extracell. Vesicles 12:e12308. doi: 10.1002/jev2.12308 36739598 PMC9899444

[B28] Kirchner S. Ignatova Z. (2015). Emerging roles of tRNA in adaptive translation, signalling dynamics and disease. Nat. Rev. Genet. 16, 98–112. doi: 10.1038/nrg3861 25534324

[B29] Klein C. Westenberger A. (2012). Genetics of Parkinson's disease. Cold Spring Harb. Perspect. Med. 2:a008888. doi: 10.1101/cshperspect.a008888 22315721 PMC3253033

[B30] Lashuel H. A. Overk C. R. Oueslati A. Masliah E. (2013). The many faces of α-synuclein: from structure and toxicity to therapeutic target. Nat. Rev. Neurosci. 14, 38–48. doi: 10.1038/nrn3406 23254192 PMC4295774

[B31] Lau Y. Patki G. Das-Panja K. Le W. Ahmad S. O. (2011). Neuroprotective effects and mechanisms of exercise in a chronic mouse model of Parkinson's disease with moderate neurodegeneration. Eur. J. Neurosci. 33, 1264–1274. doi: 10.1111/j.1460-9568.2011.07626.x 21375602 PMC3079264

[B32] Lee M. J. Park J. Ryu D. Yoo D. Cheon S. (2025). Factors associated with the response to exercise in patients with Parkinson's disease. J. Mov. Disord. 18:308. doi: 10.14802/jmd.25068 40375631 PMC12580730

[B33] Li H. Liu C. Li R. Zhou L. Ran Y. Yang Q. . (2024). AARS1 and AARS2 sense L-lactate to regulate cGAS as global lysine lactyltransferases. Nature 634, 1229–1237. doi: 10.1038/s41586-024-07992-y 39322678

[B34] Lin Y. Wang J. Zhuang X. Zhao Y. Wang W. Wang D. . (2024). Queuine ameliorates impaired mitochondrial function caused by mt-tRNAAsn variants. J. Transl. Med. 22:780. doi: 10.1186/s12967-024-05574-0 39175050 PMC11340107

[B35] Madrer N. Vaknine-Treidel S. Zorbaz T. Tzur Y. Bennett E. R. Drori P. . (2025). Pre-symptomatic Parkinson's disease blood test quantifying repetitive sequence motifs in transfer RNA fragments. Nat. Aging 5, 868–882. doi: 10.1038/s43587-025-00851-z 40216989 PMC12092246

[B36] Magee R. Londin E. Rigoutsos I. (2019). TRNA-derived fragments as sex-dependent circulating candidate biomarkers for Parkinson's disease. Parkinsonism Relat. Disord. 65, 203–209. doi: 10.1016/j.parkreldis.2019.05.035 31402278

[B37] Mailing L. J. Woods J. A. Allen J. M. Buford T. W. Fields C. J. (2019). Exercise and the gut microbiome: a review of the evidence, potential mechanisms, and implications for human health. Exerc. Sport Sci. Rev. 47, 75–85. doi: 10.1249/JES.0000000000000183 30883471

[B38] Mick E. Titov D. V. Skinner O. S. Sharma R. Jourdain A. A. Mootha V. K. (2020). Distinct mitochondrial defects trigger the integrated stress response depending on the metabolic state of the cell. ELife 9:e49178. doi: 10.7554/eLife.49178 32463360 PMC7255802

[B39] Morris R. C. Elliott M. S. (2001). Queuosine modification of tRNA: a case for convergent evolution. Mol. Genet. Metab. 74, 147–159. doi: 10.1006/mgme.2001.3216 11592812

[B40] Motorin Y. Helm M. (2022). RNA nucleotide methylation: 2021 update. Wiley Interdiscip. Rev. RNA 13:e1691. doi: 10.1002/wrna.1691 34913259

[B41] Müller M. Legrand C. Tuorto F. Kelly V. P. Atlasi Y. Lyko F. . (2019). Queuine links translational control in eukaryotes to a micronutrient from bacteria. Nucleic Acids Res. 47, 3711–3727. doi: 10.1093/nar/gkz063 30715423 PMC6468285

[B42] Nedialkova D. D. Leidel S. A. (2015). Optimization of codon translation rates via tRNA modifications maintains proteome integrity. Cell 161, 1606–1618. doi: 10.1016/j.cell.2015.05.022 26052047 PMC4503807

[B43] Ni A. Ernst C. (2022). Evidence that substantia nigra pars compacta dopaminergic neurons are selectively vulnerable to oxidative stress because they are highly metabolically active. Front. Cell Neurosci. 16:826193. doi: 10.3389/fncel.2022.826193 35308118 PMC8931026

[B44] O'Brien K. Breyne K. Ughetto S. Laurent L. C. Breakefield X. O. (2020). RNA delivery by extracellular vesicles in mammalian cells and its applications. Nat. Rev. Mol. Cell Biol. 21, 585–606. doi: 10.1038/s41580-020-0251-y 32457507 PMC7249041

[B45] Oliveira G. P. J. Porto W. F. Palu C. C. Pereira L. M. Petriz B. Almeida J. A. . (2018). Effects of acute aerobic exercise on rats serum extracellular vesicles diameter, concentration and small RNAs content. Front. Physiol. 9:532. doi: 10.3389/fphys.2018.00532 29881354 PMC5976735

[B46] Pakos-Zebrucka K. Koryga I. Mnich K. Ljujic M. Samali A. Gorman A. M. (2016). The integrated stress response. EMBO Rep. 17, 1374–1395. doi: 10.15252/embr.201642195 27629041 PMC5048378

[B47] Paldor I. Madrer N. Vaknine Treidel S. Shulman D. Greenberg D. S. Soreq H. (2023). Cerebrospinal fluid and blood profiles of transfer RNA fragments show age, sex, and Parkinson's disease-related changes. J. Neurochem. 164, 671–683. doi: 10.1111/jnc.15723 36354307

[B48] Peng G. Zhang Y. Wang Q. Li Q. Xu H. Wang E. . (2021). The human tRNA taurine modification enzyme GTPBP3 is an active GTPase linked to mitochondrial diseases. Nucleic Acids Res. 49, 2816–2834. doi: 10.1093/nar/gkab104 33619562 PMC7969015

[B49] Peters M. Hegelmaier T. Wegner F. Höllerhage M. Ye L. Niesmann C. . (2025). The role of the gut microbiome in the progression of Parkinson's disease: a systematic review of patient cohorts. J. Neurol. 273:8. doi: 10.1007/s00415-025-13545-8 41351765 PMC12681464

[B50] Petzinger G. M. Fisher B. E. McEwen S. Beeler J. A. Walsh J. P. Jakowec M. W. (2013). Exercise-enhanced neuroplasticity targeting motor and cognitive circuitry in Parkinson's disease. Lancet Neurol. 12, 716–726. doi: 10.1016/S1474-4422(13)70123-6 23769598 PMC3690528

[B51] Pizzo E. Sarcinelli C. Sheng J. Fusco S. Formiggini F. Netti P. . (2013). Ribonuclease/angiogenin inhibitor 1 regulates stress-induced subcellular localization of angiogenin to control growth and survival. J Cell Sci. 126(Pt 18), 4308–4319. doi: 10.1242/jcs.134551 23843625 PMC3772394

[B52] Poewe W. Seppi K. Tanner C. M. Halliday G. M. Brundin P. Volkmann J. . (2017). Parkinson disease. Nat. Rev. Dis. Primers 3:17013. doi: 10.1038/nrdp.2017.13 28332488

[B53] Prehn J. H. M. Jirström E. (2020). Angiogenin and tRNA fragments in Parkinson's disease and neurodegeneration. Acta Pharmacol. Sin. 41, 442–446. doi: 10.1038/s41401-020-0375-9 32144338 PMC7470775

[B54] Radak Z. Chung H. Y. Koltai E. Taylor A. W. Goto S. (2008). Exercise, oxidative stress and hormesis. Ageing Res Rev. 7, 34–42. doi: 10.1016/j.arr.2007.04.004 17869589

[B55] Rashad S. (2025). Queuosine tRNA modification: connecting the microbiome to the translatome. BioEssays 47:e202400213. doi: 10.1002/bies.202400213 39600051 PMC11755703

[B56] Rashad S. Al-Mesitef S. Mousa A. Zhou Y. Ando D. Sun G. . (2024). Translational response to mitochondrial stresses is orchestrated by tRNA modifications. bioRxiv [Preprint]. doi: 10.1101/2024.02.14.580389 38405984 PMC10888749

[B57] Romano S. Savva G. M. Bedarf J. R. Charles I. G. Hildebrand F. Narbad A. (2021). Meta-analysis of the Parkinson's disease gut microbiome suggests alterations linked to intestinal inflammation. NPJ Parkinsons Dis. 7:27. doi: 10.1038/s41531-021-00156-z 33692356 PMC7946946

[B58] Ryan L. Rubinsztein D. C. (2024). The autophagy of stress granules. FEBS Lett. 598, 59–72. doi: 10.1002/1873-3468.14787 38101818

[B59] Sampson T. R. Debelius J. W. Thron T. Janssen S. Shastri G. G. Ilhan Z. E. . (2016). Gut microbiota regulate motor deficits and neuroinflammation in a model of Parkinson's disease. Cell 167, 1469.e12–1480.e12. doi: 10.1016/j.cell.2016.11.018 27912057 PMC5718049

[B60] Scarpulla R. C. (2011). Metabolic control of mitochondrial biogenesis through the PGC-1 family regulatory network. Biochim. Biophys. Acta 1813, 1269–1278. doi: 10.1016/j.bbamcr.2010.09.019 20933024 PMC3035754

[B61] Schapira A. H. V. (2007). Mitochondrial dysfunction in Parkinson's disease. Cell Death Differ. 14, 1261–1266. doi: 10.1038/sj.cdd.4402160 17464321

[B62] Scheperjans F. Aho V. Pereira P. A. B. Koskinen K. Paulin L. Pekkonen E. . (2015). Gut microbiota are related to Parkinson's disease and clinical phenotype. Mov. Disord. 30, 350–358. doi: 10.1002/mds.26069 25476529

[B63] Shan W. P. Yan S. L. Guo Y. Y. Yang H. K. Wang J. C. Xiang J. (2025). Aerobic exercise and gut flora: a key link to improved cognitive impairment in mice with Parkinson's disease. Front. Aging Neurosci. 17:1630003. doi: 10.3389/fnagi.2025.1630003 41195080 PMC12583108

[B64] Sievers K. Neumann P. Sušac L. Da Vela S. Graewert M. Trowitzsch S. . (2024). Structural and functional insights into tRNA recognition by human tRNA guanine transglycosylase. Structure 32, 316.e5–327.e5. doi: 10.1016/j.str.2023.12.006 38181786

[B65] Sievers K. Welp L. Urlaub H. Ficner R. (2021). Structural and functional insights into human tRNA guanine transglycosylase. RNA Biol. 18, 382–396. doi: 10.1080/15476286.2021.1950980 PMC8677009 34241577

[B66] Sobala A. Hutvagner G. (2013). Small RNAs derived from the 5' end of tRNA can inhibit protein translation in human cells. RNA Biol. 10, 553–563. doi: 10.4161/rna.24285 23563448 PMC3710361

[B67] Spillantini M. Schmidt M. Lee V. Y. Trojanowski J. Q. Jakes R. Goedert M. (1997). α-Synuclein in Lewy bodies. Nature 388, 839–840. doi: 10.1038/42166 9278044

[B68] Surmeier D. J. Obeso J. A. Halliday G. M. (2017). Selective neuronal vulnerability in Parkinson disease. Nat. Rev. Neurosci. 18, 101–113. doi: 10.1038/nrn.2016.178 28104909 PMC5564322

[B69] Suzuki T. Yashiro Y. Kikuchi I. Ishigami Y. Saito H. Matsuzawa I. . (2020). Complete chemical structures of human mitochondrial tRNAs. Nat. Commun. 11:4269. doi: 10.1038/s41467-020-18068-6 32859890 PMC7455718

[B70] Tuorto F. Legrand C. Cirzi C. Federico G. Liebers R. Müller M. . (2018). Queuosine-modified tRNAs confer nutritional control of protein translation. EMBO J. 37:e99777. doi: 10.15252/embj.201899777 30093495 PMC6138434

[B71] Turchinovich A. Tonevitsky A. G. Burwinkel B. (2016). Extracellular miRNA: a collision of two paradigms. Trends Biochem. Sci. 41, 883–892. doi: 10.1016/j.tibs.2016.08.004 27597517

[B72] Valadi H. Ekström K. Bossios A. Sjöstrand M. Lee J. J. Lötvall J. O. (2007). Exosome-mediated transfer of mRNAs and microRNAs is a novel mechanism of genetic exchange between cells. Nat. Cell Biol. 9, 654–659. doi: 10.1038/ncb1596 17486113

[B73] Veldkamp K. I. Schootemeijer S. Joosten H. Bloem B. R. Evers L. J. W. de Vries N. M. (2025). Cardiorespiratory response to exercise in Parkinson's disease: associations with autonomic dysfunction and physical activity. Mov. Disord. Clin Pract. 12, 1882–1890. doi: 10.1002/mdc3.70172 40485648 PMC12625118

[B74] Wang X. Matuszek Z. Huang Y. Parisien M. Dai Q. Clark W. . (2018). Queuosine modification protects cognate tRNAs against ribonuclease cleavage. RNA 24, 1305–1313. doi: 10.1261/rna.067033.118 29970597 PMC6140461

[B75] Watanabe H. Dijkstra J. M. Nagatsu T. (2024). Parkinson's disease: cells succumbing to lifelong dopamine-related oxidative stress and other bioenergetic challenges. Int. J. Mol. Sci. 25:2009. doi: 10.3390/ijms25042009 38396687 PMC10888576

[B76] Weber D. Ferrario P. G. Bub A. (2025). Exercise intensity determines circulating levels of Lac-Phe and other exerkines: a randomized crossover trial. Metabolomics 21:63. doi: 10.1007/s11306-025-02260-0 40335829 PMC12058925

[B77] Wei Q. Wang H. Liu Y. Wang F. Wu X. Xu C. . (2025). Aerobic exercise improves clinical symptoms in people with Parkinson's disease and its potential mechanism. Front. Neurol. 16:1658162. doi: 10.3389/fneur.2025.1658162 41111965 PMC12527866

[B78] Wiener D. Schwartz S. (2021). The epitranscriptome beyond m6A. Nat. Rev. Genet. 22, 119–131. doi: 10.1038/s41576-020-00295-8 33188361

[B79] Wong Y. C. Krainc D. (2017). α-synuclein toxicity in neurodegeneration: mechanism and therapeutic strategies. Nat. Med. 23, 1–13. doi: 10.1038/nm.4269 28170377 PMC8480197

[B80] Yamasaki S. Ivanov P. Hu G. F. Anderson P. (2009). Angiogenin cleaves tRNA and promotes stress-induced translational repression. J. Cell Biol. 185, 35–42. doi: 10.1083/jcb.200811106 19332886 PMC2700517

[B81] Yasukawa T. Suzuki T. Ishii N. Ohta S. Watanabe K. (2001). Wobble modification defect in tRNA disturbs codon-anticodon interaction in a mitochondrial disease. EMBO J. 20, 4794–4802. doi: 10.1093/emboj/20.17.4794 11532943 PMC125593

[B82] Ye Y. Xu J. Shen H. Yu Z. Chen G. (2024). Neuroprotective effects of hydrogen sulfide in Parkinson's disease. Med Gas Res. 14, 145–148. doi: 10.4103/2045-9912.385945 40232693 PMC466979

[B83] Young S. K. Wek R. C. (2016). Upstream open reading frames differentially regulate gene-specific translation in the integrated stress response. J. Biol. Chem. 291, 16927–16935. doi: 10.1074/jbc.R116.733899 27358398 PMC5016099

[B84] Zhang W. Lahry K. Cipurko D. Huang S. Zbihley O. Sevilleja A. M. . (2025). Two microbiome metabolites compete for tRNA modification to impact mammalian cell proliferation and translation quality control. Nat. Cell Biol. 27, 1812–1826. doi: 10.1038/s41556-025-01750-6 40957911 PMC13267997

[B85] Zheng B. Liao Z. Locascio J. J. Lesniak K. A. Roderick S. S. Watt M. L. . (2010). PGC-1α, a potential therapeutic target for early intervention in Parkinson's disease. Sci. Transl. Med. 2:52ra73. doi: 10.1126/scitranslmed.3001059 20926834 PMC3129986

